# GASZ Is Essential for Male Meiosis and Suppression of Retrotransposon Expression in the Male Germline

**DOI:** 10.1371/journal.pgen.1000635

**Published:** 2009-09-04

**Authors:** Lang Ma, Gregory M. Buchold, Michael P. Greenbaum, Angshumoy Roy, Kathleen H. Burns, Huifeng Zhu, Derek Y. Han, R. Alan Harris, Cristian Coarfa, Preethi H. Gunaratne, Wei Yan, Martin M. Matzuk

**Affiliations:** 1 Department of Pathology, Baylor College of Medicine, Houston, Texas, United States of America; 2 Department of Molecular and Cellular Biology, Baylor College of Medicine, Houston, Texas, United States of America; 3 Department of Pathology, The Johns Hopkins School of Medicine, Baltimore, Maryland, United States of America; 4 Department of Biology and Biochemistry, University of Houston, Houston, Texas, United States of America; 5 Department of Molecular and Human Genetics, Baylor College of Medicine, Houston, Texas, United States of America; 6 Human Genome Sequencing Center, Baylor College of Medicine, Houston, Texas, United States of America; 7 Department of Physiology and Cell Biology, University of Nevada School of Medicine, Reno, Nevada, United States of America; University of California San Francisco, United States of America

## Abstract

Nuage are amorphous ultrastructural granules in the cytoplasm of male germ cells as divergent as *Drosophila*, *Xenopus*, and *Homo sapiens*. Most nuage are cytoplasmic ribonucleoprotein structures implicated in diverse RNA metabolism including the regulation of PIWI-interacting RNA (piRNA) synthesis by the PIWI family (i.e., MILI, MIWI2, and MIWI). MILI is prominent in embryonic and early post-natal germ cells in nuage also called germinal granules that are often associated with mitochondria and called intermitochondrial cement. We find that GASZ (Germ cell protein with Ankyrin repeats, Sterile alpha motif, and leucine Zipper) co-localizes with MILI in intermitochondrial cement. Knockout of *Gasz* in mice results in a dramatic downregulation of MILI, and phenocopies the zygotene–pachytene spermatocyte block and male sterility defect observed in MILI null mice. In *Gasz* null testes, we observe increased hypomethylation and expression of retrotransposons similar to MILI null testes. We also find global shifts in the small RNAome, including down-regulation of repeat-associated, known, and novel piRNAs. These studies provide the first evidence for an essential structural role for GASZ in male fertility and epigenetic and post-transcriptional silencing of retrotransposons by stabilizing MILI in nuage.

## Author Summary

Many aspects of RNA processing are essential for or prominent in the differentiation of germ cells. Some RNA metabolism in animal germ cells is associated with physical structures surrounding the cell nucleus called nuage. Nuage has a distinct granular appearance prior to the meiotic divisions with unclear functions. We have identified a protein called GASZ, which plays a structural role in this early nuage. In mice lacking GASZ, retrotransposons—endogenous viral-like particles—become released from their typical repressed state in the germline by the loss of small RNAs called piRNAs, resulting in DNA damage and delayed germ cell maturation. Protection of the germline from genetic intruders may require the association of piRNA-synthesizing enzymes and other components of this nuage structure through direct or indirect associations with GASZ. Mutations in GASZ and other nuage components may contribute to infertility in men who do not produce spermatozoa.

## Introduction

The differentiation program of the germline is distinct from somatic cells in that resetting of the epigenome by demethylation of DNA and histones must take place for proper post-fertilization development of the embryo [1]. DNA demethylation occurs during primordial germ cell (PGC) migration as part of their normal development [2]. Different elements within the genome are remethylated at distinct time windows in a sex-specific fashion. Remethylation of retrotransposons occurs in the male germline at embryonic day 17.5 (E17.5) and in the female germline during postnatal oocyte maturation [1]. The resetting of the epigenetic state of the germline followed by the acquisition of male-specific methylation imprints, while a necessary component for post-fertilization development, exposes the germline to potential risk from retrotransposon mobilization [3]. Insects and mammals resolve this problem through the action of several classes of small RNAs including piRNAs (∼27 nt PIWI family-interacting RNAs) [4]–[9]. Two classes of piRNAs, repeat-associated piRNAs and non-repeat-associated piRNAs based on their similarity to retrotransposons, are present in the germline of animals as primitive as sponges [10]. Repeat-associated piRNAs limit expression of retrotransposons at the post-transcriptional level and through epigenetic silencing by the recruitment of DNA methyltransferases including DNMT3A and DNMT3L [11]–[15]. In the absence of these small RNAs, retrotransposon expression is dramatically increased in the germline, leading to DNA damage and cell death.

Regulation of retrotransposon repression is coordinated by proteins in spatially specialized compartments of ribonucleoprotein-rich structures called nuage. According to the nomenclature proposed by Chuma et al. [16], embryonic prospermatogonia, postnatal spermatogonia and spermatocytes possess a form of nuage appearing as perinuclear granules transiently associated with mitochondria and thus termed intermitochondrial cement. In contrast, a single large granule of nuage, present in post-meiotic spermatids is called the chromatoid body [17],[18]. Multiple proteins have been localized by electron microscopy to both intermitochondrial cement and the chromatoid body including mouse VASA homolog (MVH; also called DDX4 or DEAD-box polypeptide 4), tudor-domain containing 1 (TDRD1), tudor-domain containing 6 (TDRD6), and tudor-domain containing 7 (TDRD7) [19]–[21]. The chromatoid body is not believed to arise merely by coalescence of intermitochondrial cement granules; however, to date there are no examples of proteins localized by electron microscopy specifically to the intermitochondrial cement but absent from the chromatoid body.

Nuage are proposed sites for multiple RNA processing events including translational repression, RNA-mediated gene silencing, mRNA degradation, and nonsense-mediated mRNA decay [22]. A number of germ cell-specific mRNAs display translational repression with a lag of up to a week between their transcription and translation [23],[24]. The evidence for nuage regulation of mRNA is strongest for the chromatoid body. Translationally regulated mRNAs such as transition protein 2 (*Tnp2*) have been localized to the chromatoid body [25]. DEAD box helicases, including MVH/DDX4 and DDX25, which can unwind RNA *in vitro*, are localized to the chromatoid body [26]–[30]. MicroRNAs (∼22 nt non-coding RNAs) and components of the RNA-Induced Silencing Complex (RISC) machinery including Dicer, Argonaute 2 (AGO2), and Argonaute 3 (AGO3) with demonstrated *in vitro* endonuclease activity, localize to the chromatoid body where they may function in translational control and mRNA stability, but their potential association with the intermitochondrial cement is not described [22],[31]. The chromatoid body also contains MILI and MIWI RNA endonucleases that generate piRNAs [32],[33]. Most MIWI-associated mRNAs in spermatids are associated with the RNP fraction with a smaller number associated with polysomes suggestive of a function in translational control [34].

Prior to the meiotic divisions, the role of nuage in mRNA metabolism in primordial germ cells, spermatogonia, and spermatocytes is unknown. Whereas MILI is present throughout this period, MIWI2 is restricted to nuage granules in embryonic testes, and MIWI is present in those of pachytene spermatocytes [13], [35]–[37]. The localization of PIWI family proteins to the intermitochondrial cement has not been clearly defined, although MILI interacts with the intermitochondrial cement protein TDRD1, MVH and other nuage proteins [33],[38]. Maelstrom (MAEL), the putative 3′–5′ endonuclease for piRNA 3′ end formation, is also associated with nuage in mammals [39]–[41]. Thus, multiple components necessary for piRNA generation are connected physically to nuage.

Genetic evidence supports a conserved requirement for proper nuage assembly in retrotransposon control in the germline. In *Drosophila*, mutants of PIWI, aubergine (AUB), AGO3, the RNA helicases VASA and armitage (ARMI), and TUDOR-domain containing proteins krimper (KRIMP) and spindle-E (SPN-E) have defects in piRNA synthesis and retrotransposon control. In *Drosophila*, the interaction of two PIWI family members with opposite strand polarity during nuage assembly has also been proposed to facilitate the “ping-pong” mechanism of amplification necessary for retrotransposon inhibition [42]. piRNA defects and derepression of retrotransposons in germ cells also occurs in zebrafish ZILI and ZIWI mutants [43],[44]. Consistent with a key role of these nuage-associated proteins in the mammalian male germline, knockouts of *Mvh*, *Mael*, *Mili*, and *Miwi2* block at the spermatocyte stage, while knockouts of TDRD1, TDRD6, and MIWI disrupt spermatogenesis at the spermatid stage. With the exception of MVH, which has not been assessed for these defects, all other nuage mutants with a spermatocyte arrest have defects in retrotransposon regulation and piRNA production [7], [20], [35], [38], [45]–[48].

GASZ is a 475 amino acid Germ cell-specific protein with four Ankyrin repeats, a Sterile alpha motif, and a basic leucine Zipper domain [49] that is conserved across vertebrate evolution in amphibians, fish, birds, and mammals [50]. Our previous studies have shown that GASZ localizes to the Balbiani body, a nuage structure in *Xenopus laevis* oocytes. The high degree of evolutionary conservation of GASZ in vertebrates and the potential localization of GASZ to a conserved germline-specific structure stimulated our interest to determine the expression and essential roles of GASZ in nuage and its germline function in mammals.

## Results

### Knockout of GASZ Results in Male Sterility

**Figure 1:**
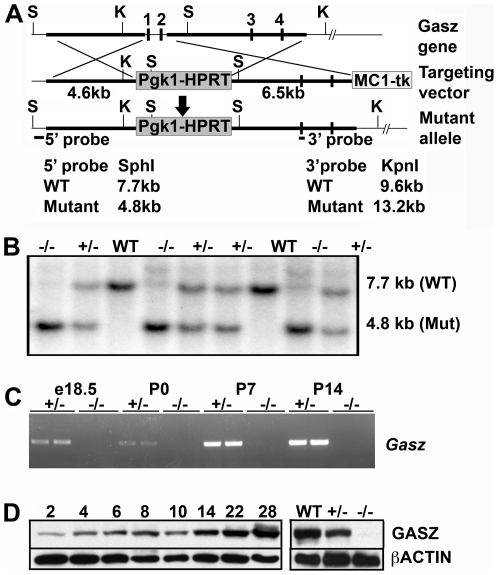
Targeting of the *Gasz* allele and generation of *Gasz* mutant mice. (A) Schematic representation of the *Gasz* gene, structure of the targeting vector, and the resultant mutant allele. Genomic DNA fragments used as 5′ and 3′ homology arms in the targeting vector are indicated by thick lines. Exons 1 and 2 (which encode the *Gasz* transcriptional start site and the initiation ATG codon) are replaced by a *PgkHPRT* expression cassette (shaded boxes). The 5′ and 3′ probes (filled boxes) used for Southern blots are indicated. (S) Sph I, (K) Kpn I. The MC1*tk* expression cassette was used for negative selection. (B) Southern blot analysis of genomic DNA derived from a litter from *Gasz* ^+/−^ (+/−) intercrosses. Similar percentages of male and female mice were genotyped as *Gasz* homozygous null (−/−). The 5′ probe hybridizes to 7.7 kb (wild-type, WT) and 4.8 kb (mutant, Mut) SphI fragments. (C) RT-PCR analysis of Gasz expression in *Gasz^+/−^* (+/−) and *Gasz^−/−^* (−/−) testes from E18.5 to post-natal day 14 demonstrating Gasz expression in embryonic testes and the lack of *Gasz* mRNA in null testes. (D) Western blot analysis of wild type testis samples from different time-points as well as 6-week-old *Gasz* WT, +/−, and −/− mice using a polyclonal antibody to GASZ (*Upper*) or an antibody to β-actin as a control for sample loading (*Lower*). GASZ protein is detected as early as postnatal day 2 and peak abundance is reached after 14 days of age. Absence of the GASZ protein in *Gasz* ^−/−^ testes confirmed that the *Gasz* ^−/−^ mutation was null.

To define the roles of GASZ in mammals, a null mutation in *Gasz* was generated (Figure 1). *Gasz* ^+/−^ mice were viable and produced pups (1.00±0.01 litters/month; 8.25±0.29 pups/litter, n = 10) whose genotypes were consistent with Mendelian ratios (25.9% WT, 48.5% *Gasz* ^+/−^, 25.7% *Gasz* ^−/−^; n = 495) indicating that GASZ is not essential for embryogenesis. Whereas *Gasz* ^−/−^ females were fertile (0.95±0.02 litters/month; 6.16±0.32 pups/litter), *Gasz* ^−/−^ males were sterile. Furthermore, although GASZ is a maternal effect protein [49], observed in early preimplantation embryos, the viability of offspring from Gasz null females (i.e., oocytes lacking *Gasz* mRNA) indicates that maternal GASZ is also not required.

### *Gasz* ^−/−^ Males Demonstrate a Defect in Meiosis

**Figure 2:**
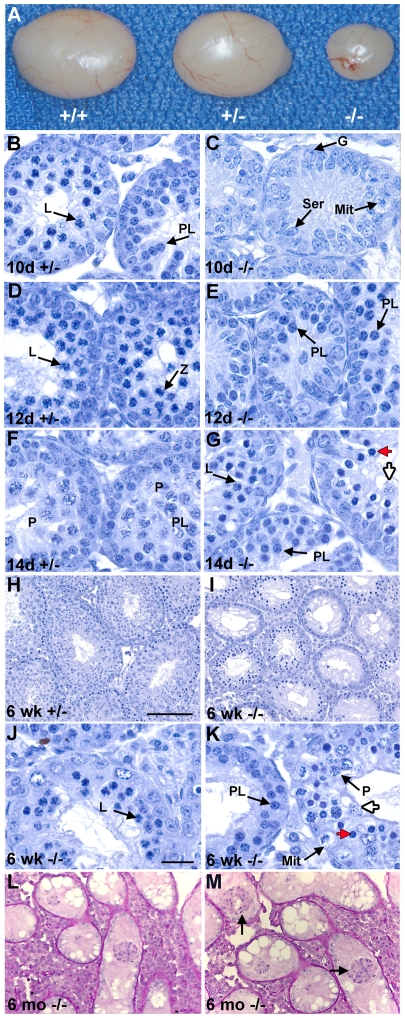
Gross and histological analysis of postnatal testes. (A) Gross analysis of testes from 7-week-old littermates. (B–M) Histological analysis of testes of *Gasz* ^+/−^ and *Gasz* ^−/−^ mice. (M) is a higher power magnification of (L) to show the lack of germ cells attached to the base of the tubule and sloughing germ cells in the lumen. G, spermatogonia; L, leptotene spermatocytes; M, meiotically dividing spermatocytes; Mit, mitotically dividing spermatogonia; P, pachytene spermatocytes, PL, preleptotene spermatocytes; Ser, Sertoli cells; Z, zygotene spermatocytes; dying spermatocytes with compact chromatin (red arrowhead); and with diffuse chromatin (open arrowhead); sloughing germ cells (black arrowheads). [Scale bars: 100 µm (B–C), 20 µm (D–K).]

To define the cause of the infertility in *Gasz* ^−/−^ males, postnatal testes were analyzed grossly and histologically (Figure 2). Testes from *Gasz* ^−/−^ males were significantly smaller (*P<*0.0001) than *Gasz* ^+/−^ or WT littermates (Figure 1A). Six-week-old *Gasz* ^−/−^ testes (19.2±1.07 mg; *n* = 20) were ∼20% of the weight of WT (89.9±5.68 mg; *n* = 20) and *Gasz* ^+/−^ (85.8±5.50 mg; *n* = 20) testes. Whereas WT and *Gasz* ^+/−^ testes from adult males demonstrated robust spermatogenesis (Figure 2H), the seminiferous tubules of 6-week-old *Gasz* ^−/−^ testes showed markedly reduced spermatocytes, no post-meiotic spermatids or spermatozoa, and significant vacuolization (Figure 2I–2K). The most mature meiotic cells in seminiferous tubules from stages VII–XII were early meiotic germ cells while those from stages I–VI showed a mixture of degenerating spermatocytes.

*Gasz* ^−/−^ and *Gasz* ^+/−^ testes at postnatal day 5 (P5) were similar at the gross and histologic levels (Figure S1). At postnatal day 10 (P10), the composition of *Gasz* ^−/−^ testes (Figure 2C) had not changed substantially in composition, whereas *Gasz* ^+/−^ testes (Figure 2B) had advanced to contain preleptotene and leptotene spermatocytes. At P12, *Gasz* ^−/−^ testes (Figure 2E) contained predominantly spermatogonia with only 25% of the tubules containing preleptotene spermatocytes but no leptotene spermatocytes as compared with *Gasz* ^+/−^ testes where more advanced zygotene spermatocytes were present (Figure 2D). By P14, *Gasz* ^−/−^ testes displayed early pachytene germ cell loss due to apoptosis (Figure S3), with the most advanced germ cells being zygotene spermatocytes (Figure 2G), while *Gasz* ^+/−^ testes (Figure 2F) had advanced to the mid-pachytene stage. Thus, histological analysis supports a consistent delay in *Gasz^−/−^* spermatogenesis beginning at meiotic prophase. *Gasz^−/−^* spermatogenesis fails at an identical point in juveniles and young adults. However, by 6 months of age, there are few germ cells in the *Gasz^−/−^* testes with many tubules displaying a Sertoli cell only phenotype (Figure 2L and 2M).

To further confirm our histological findings, we analyzed several genes that are expressed in spermatocytes (Figure S2 and Figure S3). γH2AX is expressed from late spermatogonia through pachytene spermatocytes [51]. γH2AX shifts from staining autosomes to the XY body in pachytene spermatocytes. XY body staining by γH2AX was absent from *Gasz* ^−/−^ testes, in contrast to *Gasz* ^+/−^ testes (Figure S2). Instead, γH2AX labeled autosomes similar to *Gasz* ^+/−^ early spermatocytes or dying spermatocytes with increased intensity. Our findings are reminiscent of MILI, MIWI2, and MVH knockout mouse models that demonstrate male sterility due to a zygotene:pachytene block but normal female fertility [7],[38],[45].

### GASZ Is a Component of the Intermitochondrial Cement and Co-Localizes with MILI

**Figure 3:**
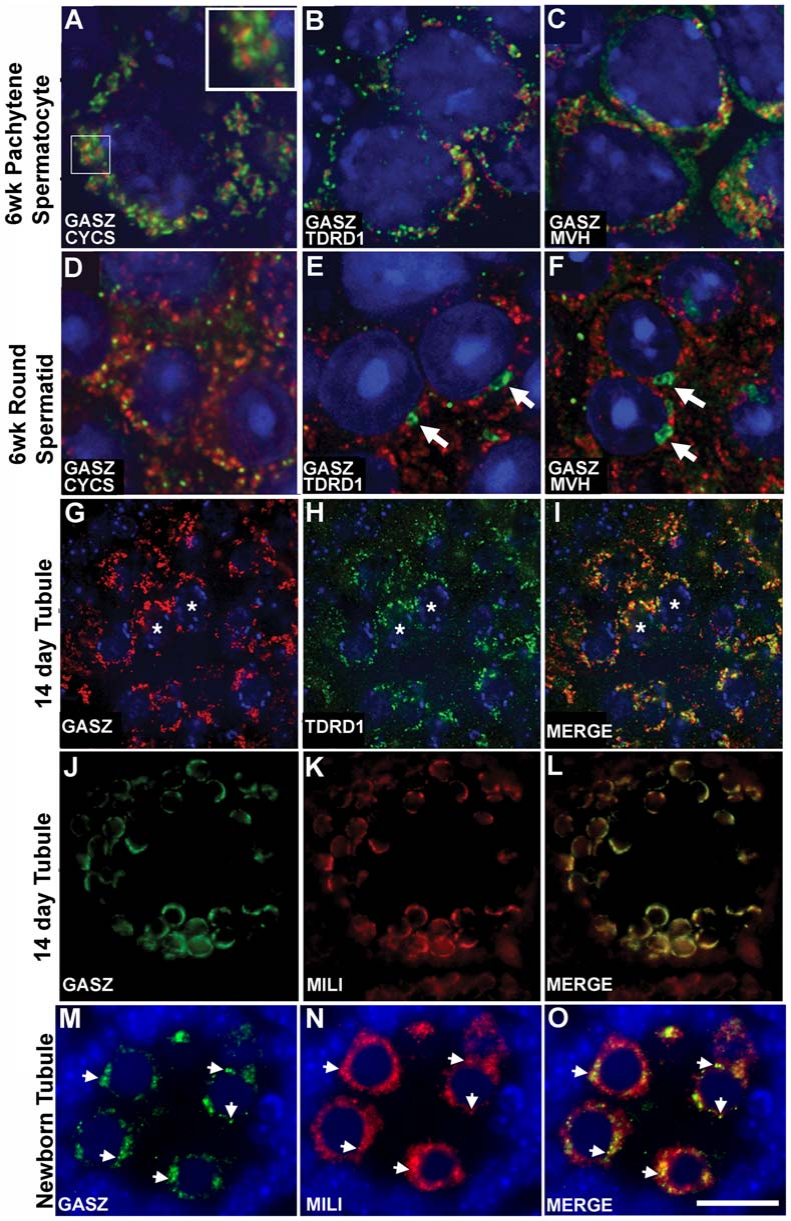
GASZ co-immunolocalization with intermitochondrial cement markers. (A–F) Immunofluorescent analysis of spermatocytes (A–C) and spermatids (D–F). (A,D) GASZ (red) localizes between mitochondrial clusters in spermatocytes using antibodies to cytochrome c (green). (A *inset*) Higher magnification. (B,E) Staining is shown for GASZ (red), TDRD1 (green). (C,F) Staining for GASZ (red) and MVH (green). Arrows in (E,F) identify the chromatoid body. Note the presence of the GASZ (red) foci and corresponding TDRD1 and MVH (green) foci in spermatocytes (A–C) but not in spermatids (D–F). (G–I) Staining is shown for GASZ (G), TDRD1 (H), and merged (I). (J–L) Staining is shown for GASZ (J), MILI (K), and merged (L). (M–O) Staining of newborn testes is shown for GASZ (M), MILI (N), and merged (O). Note the presence of the GASZ (green) foci and corresponding MILI (red) foci in gonocytes (*arrowheads*). [Scaling: 10,000×(A–F), 40X(G–L), and 400×(M–O) magnification.]

GASZ is present at low levels in the cytoplasm of type A and B spermatogonia and pre-leptotene spermatocytes, showing peak intensity in middle to late pachytene spermatocytes, and localizing to finer granules in the cytoplasm of round spermatids (Figure S4 and Figure S5). Using antibodies against mitochondrial cytochrome c, GASZ was confirmed to localize to the interstices of spermatocyte mitochondrial clusters (i.e., intermitochondrial cement) (Figure 3A and *inset*), consistent with our previous findings that GASZ localizes to nuage in frog oocytes [50]. GASZ partially co-localizes with TDRD1 and MVH in the intermitochondrial cement (Figure 3B–3I) and displays a substantial overlap with MILI in spermatocytes (Figure 3J–3L). Whereas TDRD1 and MVH relocalize to the chromatoid body in spermatids (Figure 3E and 3F), GASZ fails to do so and also shows no relationship with unclustered mitochondria (Figure 3D). In late pachytene spermatocytes MIWI and GASZ also overlap (Figure S6). MVH shows intense granular distribution in *Gasz* ^+/−^ testes but is dramatically reduced in the *Gasz* ^−/−^ spermatocytes (Figure S7).

**Figure 4:**
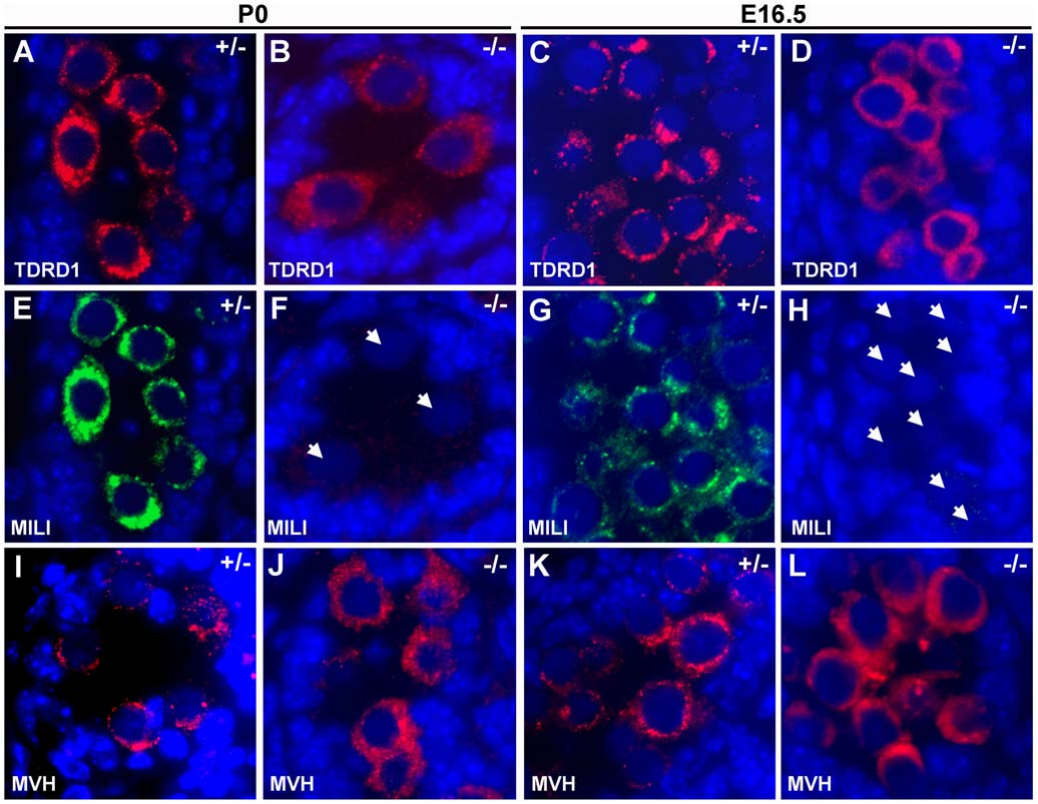
MILI is lost in embryonic and newborn testes in the absence of GASZ. Immunofluorescent analysis of *Gasz* ^+/−^ (A,C,E,G,I,K) and *Gasz^−/−^* (B,D,F,H,J,L) testes. Staining is shown for TDRD1 (A–D), MILI (E–H), and MVH (I–L). TDRD1 immunostaining is diffusely cytoplasmic in *Gasz^−/−^* newborn (B) and embryonic (D) gonocytes versus controls (A,C). Staining of the identical germ cells shows MILI is undetectable in newborn gonocytes and present in only a subset of *Gasz^−/−^* E16.5 gonocytes [*arrowheads* in (F,H)]. MVH staining is less granular in *Gasz^−/−^* (J,L) versus controls (I,K). [Scaling: 5,000×magnification.]

We also analyzed the relationship of GASZ and MILI at earlier time points to assess their interaction in immature germ cells. MILI was present in perinuclear granules in cell cycle arrested gonocytes of newborn mice (Figure 3N) in a distribution similar to that described in embryonic male germ cells [13]. There is a significant overlap between GASZ and MILI in newborn gonocytes (Figure 3M–3O). We compared the immunostaining of nuage proteins in the newborn *Gasz^−/−^* testis versus controls. TDRD1 was reduced but also diffusely cytoplasmic, failing to localize in a perinuclear granular pattern (Figure 4B). MILI was strikingly absent from the same GASZ null gonocytes (Figure 4F). MVH immunostaining also showed reduced granular localization (Figure 4J). Analysis of TDRD1 (Figure 4D) and MVH (Figure 4L) at E16.5 revealed a similar delocalization of these proteins in GASZ null gonocytes. However, MILI staining of E16.5 gonocytes (Figure 4H) was variable; in most germ cells, MILI staining was absent, whereas in only 2.25% (5 out of 222 gonocytes) granular MILI staining remained.

### GASZ Interaction Affects the Level of Intermitochondrial Cement Proteins

**Figure 5:**
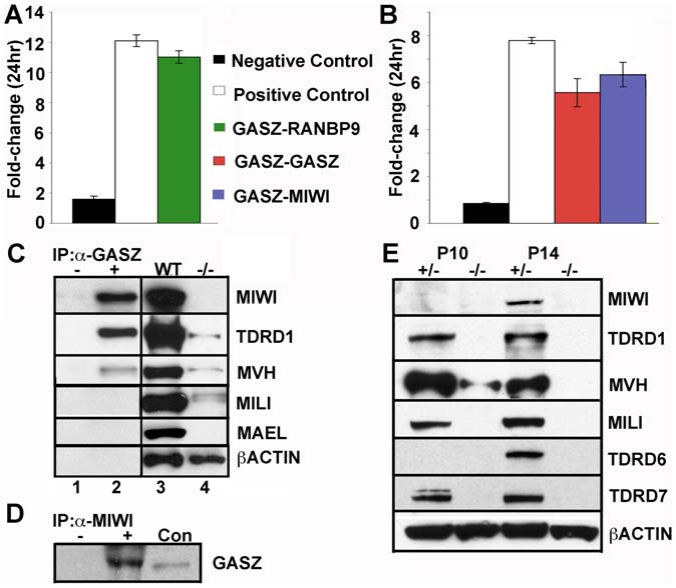
GASZ interactions and reduction of nuage proteins in *Gasz* null testes. Biosensor quantification of interaction for a full-length GASZ bait by (A) RANBP9 partial clone, and by (B) GASZ and MIWI. (C,D) GASZ co-immunoprecipitation with nuage proteins. Testicular protein lysates from 21-day-old mice were incubated with no primary antibody [lane 1, (C,D)], anti-GASZ antibody [lane 2, (C)] or anti-MIWI antibody [lane 2, (D)] to immunoprecipitate protein complexes. 10 µg of WT lysate [lane 3, (C,D)] and *Gasz^−/−^* lysate [(lane 4, (C)] were used as controls. Co-immunoprecipitating proteins were detected by western blot analysis using antibodies against MIWI, TDRD1, MVH, MILI, MAEL, TDRD6, TDRD7, or β-actin. (E) Western blot analysis of testicular protein lysates prepared from 10- and 14-day-old *Gasz^+/−^* (+/−, lanes 1 and 3) and *Gasz^−/−^* (−/−, lanes 2 and 4) mice. Antibodies against MIWI, TDRD1, MVH, MILI, TDRD6, TDRD7, or an antibody to β-actin show levels of nuage proteins are reduced in *Gasz^−/−^* testes prior to spermatocyte apoptosis.

To study GASZ protein:protein interactions, we screened a P17 testis library by yeast two-hybrid analysis using full-length GASZ as bait. We found GASZ interacts with itself and RANBP9 (Figure 5A), a known MVH interactor that also localizes to nuage and the chromatoid body [52]. MIWI, but not MILI, or MVH, also directly interacts with GASZ (Figure 5B). Antibodies to GASZ could co-immunoprecipitate MIWI, TDRD1, and MVH, but not MILI or MAEL from P21 testes (Figure 5C). Conversely, antibodies to MIWI could also co-immunoprecipitate GASZ (Figure 5D). We observed a striking reduction in the amount of multiple intermitochondrial cement proteins in *Gasz^−/−^* testis lysates at P21 as well as at times prior to the observed spermatocyte loss (Figure 5E). Multiple interactions between these nuage proteins suggest that GASZ is a component of this network. Quantitative RT-PCR showed relatively modest reduction (roughly 2-fold) in the intermitochondrial cement mRNAs in contrast to their change in protein abundance (Figure S8 and Table S1). Our findings by Western blot analysis were consistent with reduced MVH immunostaining of spermatocytes (Figure S7) and absence of MILI in gonocytes (Figure 4). We also examined newborn testes by electron microscopy for the presence of intermitochondrial cement. Although we could find nuage material associated with clustered mitochondria in most neonatal gonocytes in controls, we failed to detect this structure in *Gasz^−/−^* germ cells (Figure S9). Our results indicate that GASZ plays a key role in the formation and/or maintenance of this network of RNA processing proteins.

### Absence of GASZ Causes Hypomethylation of Retrotransposons and Their Increased Expression

**Figure 6:**
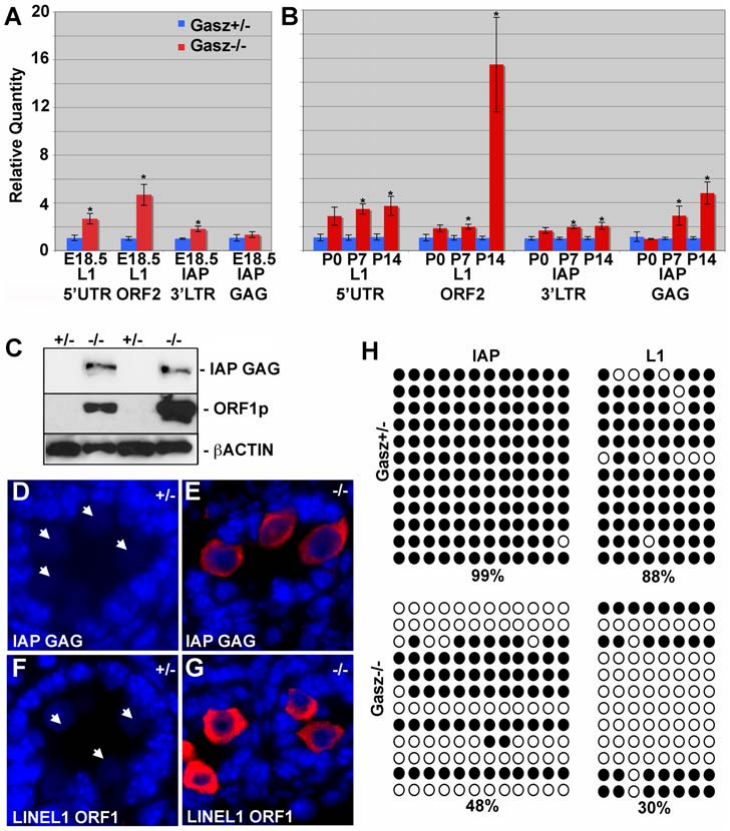
Dysregulation of transposable elements in *Gasz* null testes. (A–B) Quantitative RT-PCR analysis of transposable elements in testes from embryonic, newborn, 7- and 14-day-old mice (mean±SEM). (C) Western blot analysis of testis samples from 14-day-old mice by using anti-GAG (*Upper*), anti-ORF1 (Middle), or anti-β-actin control (*Lower*) demonstrated increased IAP GAG and LINE L1 ORF1p expression in *Gasz^−/−^* testes. (D–G) Immunofluorescent analysis of IAP GAG (D–E) and ORF1p (F–G). Robust staining of IAP GAG and ORF1p is detected in *Gasz^−/−^* gonocytes (E,G) but absent from Gasz^+/−^ controls (D,F). (H) CpG methylation analysis of IAP and LINE L1 using bisulfite-converted testicular genomic DNA. Methylated CpG dinucleotides remain unconverted as cytosine (filled circles) and unmethylated cytosines are converted to uracils and amplified as thymidines (open circles). Percentages of CG dinucleotide methylation are given.

There is increased retrotransposon transcription in MILI and MIWI2 null testes [7],[38], and dying *Gasz^−/−^* spermatocytes have a characteristic chromatin pattern similar to these knockouts. Because of these similarities and GASZ association with MIWI, we measured levels of the retrotransposons intracisternal A particle (IAP) and long interspersed nuclear element 1 (Line L1) in the testes of P14 *Gasz^−/−^* mice. Quantitative RT-PCR demonstrated up to a 15-fold increase (p<0.05) in Line L1 and up to a 4-fold increase in IAP mRNA (p<0.05) in the postnatal *Gasz^−/−^* testis as well as similar increases in embryonic testes compared to controls (Figure 6A and 6B). The largest increases were seen in the Line L1 ORF2 (encoding the reverse transcriptase and endonuclease domains) and IAP GAG mRNAs at P14. We saw even more dramatic increases in IAP GAG protein and Line L1 ORF1p. At P14, levels of IAP GAG and Line L1 ORF1p proteins are essentially undetectable in the WT samples but significantly up-regulated in the *Gasz^−/−^* testes (Figure 6C). Likewise, in gonocytes of control newborn (P0) mice, retrotransposon proteins IAP GAG and Line L1 ORF1p were undetectable but were dramatically elevated in the cytoplasm of the *Gasz^−/−^* gonocytes (Figure 6D–6G).

Consistent with compromised transposable element DNA methylation seen in MILI and MIWI2 null testes, we found significant hypomethylation of presumed germ cell-derived Line L1 and IAP sequences in *Gasz* ^−/−^ testes compared to controls at P14 (Figure 6H). Sequences demonstrating appropriate methylation in the *Gasz* knockout may reflect the presence of admixed somatic cell DNA. Thus, the delayed meiotic initiation and spermatocyte apoptosis in the *Gasz^−/−^* testes are likely secondary to abnormal derepression of retrotransposons in the male germline at the transcriptional and post-transcriptional levels.

### Suppression of PIWI-Interacting RNAs (piRNAs) in GASZ Null Testes

**Figure 7:**
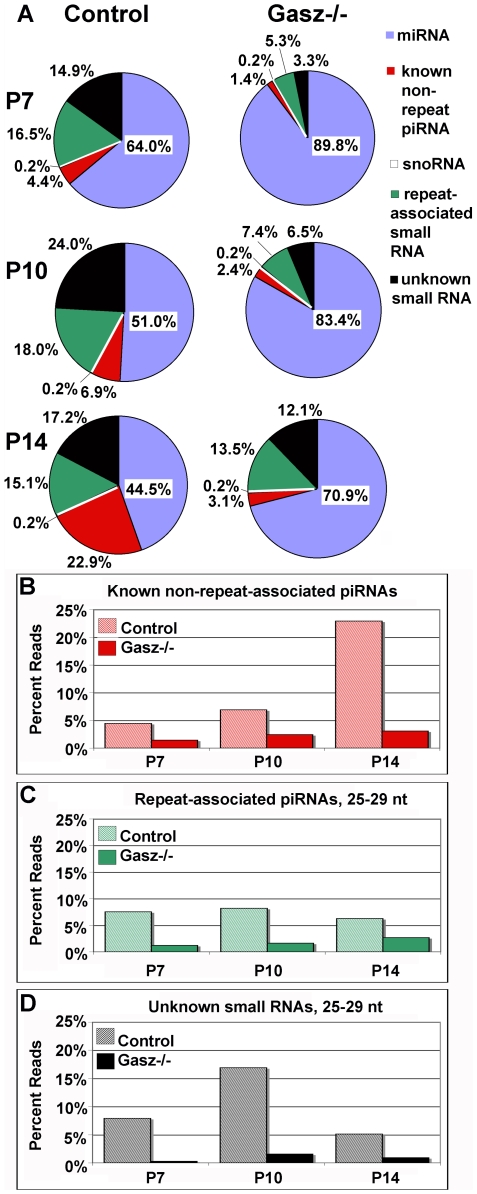
Repeat and non-repeat piRNAs regulated by GASZ. (A) Compositional analysis of small RNA populations at postnatal days 7 (P7), 10 (P10), and 14 (P14) in *Gasz* ^+/−^ (*Left*) and *Gasz* ^−/−^ (*Right*) testes with annotation of small RNA populations as described in experimental procedures. (B–D) Comparison of the relative abundance of several classes of known and putative novel piRNAs between *Gasz* ^−/−^ and control testes at postnatal days 7, 10, and 14 including known non-repeat-associated piRNAs (B), repeat-associated piRNAs (25–29 nt) (C) and unknown small RNAs (25–29 nt) (D).

Since increased retrotransposon expression in MILI and MIWI2 null germ cells has been ascribed to the loss of repression by repeat-associated RNAs, we evaluated their abundance by small RNA sequencing of P7, P10 and P14 *Gasz^+/−^* and *Gasz^−/−^* testes using the Illumina Next Generation Sequencing platform that yields over 2 million sequence reads per sample. Control testes showed an increase in pachytene piRNAs at P14, resulting in a proportional reduction in miRNA reads contributing to the overall small RNA pool. By contrast, *Gasz^−/−^* testes failed to induce piRNAs, and the small RNAome (17–40 nt) was dominated by miRNAs in *Gasz^−/−^* testes compared to controls (71% vs. 44%, Figure 7A). However, the overall miRNA profiles (the subset of miRNAs expressed and relative abundance) were strikingly similar in both genotypes suggesting that miRNA biogenesis and function are likely intact in the absence of GASZ; the relative increase in miRNA read abundance is a consequence of reduced piRNA sequences within a fixed sample of small RNA reads. MicroRNAs have been shown to be present in the chromatoid body, but based on our analysis, they are clearly present prior to the appearance of this structure. This observation suggests that the formation of the intermitochondrial cement and chromatoid body are not required for microRNA biogenesis, but might be important for their action on target mRNAs.

After excluding potential contributions to the *Gasz* null phenotype by miRNAs, we analyzed the remaining small RNAs in greater detail. This analysis revealed that most non-repeat-associated piRNAs showed a skewed distribution with 6% contributing 95–99% of the reads. There was a similar low number of known non-repeat-associated piRNAs in the control and null samples at P10 (Figure 7A), consistent with previous studies demonstrating a robust increase at P14 pachytene stage in wild-type testes [6]. Non-repeat-associated piRNAs contributed only 14% of the piRNA reads in control testes at this age. However, 99% of the 6500 non-repeat-associated piRNAs detected in control P10 testes were reduced in *Gasz^−/−^* testes, 87% to undetectable levels. The decline was even more dramatic in P14 *Gasz^−/−^* testes when many more piRNAs are produced in the control testes (Figure 7B and Table S2). Although the delayed spermatogenic development and apoptotic loss of spermatocytes could preclude the expression of these piRNAs in *Gasz^−/−^* testes, the concurrent increase in miRNAs and expression of prepachytene piRNAs, normally abundant by P8 [6], indicate that these two effects are unlikely to be the cause. Over 1500 of 1700 distinct non-repeat-associated RNAs (92%) with greater abundance at P7 versus P14 were substantially reduced, and 1418 of these (90%) displayed a sustained reduction at subsequent time-points (Table S2). Twenty-seven of the 100 small RNAs displaying a relative increase in *Gasz^−/−^* testes appeared to be variants of miRNAs or potential passenger strands, many mapping to a miRNA cluster on the X chromosome (Table S3). Two additional 22–23 nt sequences, DQ712837 mapping to Small Cajal body specific RNA 15 (*Scarna15*) and DQ688886 mapping to an intron of *1700041C02Rik*, contributed 40% of the “piRNA” reads remaining in *Gasz^−/−^* testes. The 127 nt *Scarna15* is predicted to form two stem-loops and DQ712837 derives from the stem of the second stem-loop. If we exclude these two likely misannotated Dicer-dependent small RNAs, the non-repeat-associated piRNAs reads are 99% reduced.

Control testes express an abundance of repeat-associated small RNAs compared to the *Gasz^−/−^* testes (Figure 7A and Table S4), which fall into two size categories that peak at 22 and 27 nt (Figure S10A). While there is a small peak at 22 nt in *Gasz^−/−^* testes, the 27 nt peak is essentially absent. To analyze the repeat-associated piRNAs, we excluded all sequences whose length was not 25–29 nt. Many of the 19–23 nt sequences map to SINEs (Figure S10D and Figure S10E and Table S3) and typically show less than a two-fold change. These SINE-associated small RNAs belong to a novel small RNA class that are cleaved from their precursor RNA by a Dicer-dependent but DGCR8-independent mechanism [53]. We also excluded 19–23 nt sequences that did not map to SINEs as potential Dicer-dependent endo-siRNAs (Figure S10F and Figure S10G). All 25–29 nt repeat-associated RNAs were classified as repeat-associated piRNAs (Figure 7C). These repeat-associated piRNAs map to LTRs (e.g., ERV-K IAPLTR1a_I_MM and MaLR MTA_Mm_LTR) or LINEs (L1_MM), and are 10 to 100-fold less abundant in *Gasz^−/−^* testes (Figure S10B and Figure S10C and Table S4). The majority of the 25–29 nt repeat-associated piRNAs contained a U at position 1 and an A at position 10 (Figure S10H), characteristic of participation in the “ping-pong” synthesis reaction [54]. In contrast, the 19–23 nt repeat-associated small RNAs had an initial U but a variable position 10 (Figure S10I), suggesting a lack of amplification by the “ping-pong” mechanism and confirming that these sequences are unlikely to be synthesized by the piRNA machinery. Subsequent analysis showed that the biggest difference in repeat-associated piRNAs occurred at P7 when many more elements were affected. Thus, the defect in repeat-associated piRNA production preceding delayed meiotic prophase initiation in *Gasz^−/−^* testes is a cause rather than an effect of this process.

Initially, 25% of the small RNAs sequences (>1 million reads) in control testes were unclassified. The majority of the unclassified sequences were 25–29 nt (Figure 7D and Figure S10J), but a 19–23 nt class was also present (Figure S10K). The majority of these unclassified RNAs possess a 5′ U but a variable 10^th^ position (Figure S10L). Because of their similarity to piRNAs, we term the 25–29 nt category of unclassified sequences as “putative piRNAs” while the smaller size category called “unknown small RNAs” may contain novel miRNAs or other Dicer-dependent small RNAs. “Putative piRNA” reads were reduced compared to controls at P7 (7.9% versus 0.29%), P10 (1.6% versus 16.9%) and P14 (5.10% versus 0.91%) (Figure 7D). These findings indicate that GASZ plays a major role prior to the pachytene stage in facilitating the production of multiple types of piRNAs, including those associated with repeats involved in regulation of retrotransposons.

## Discussion

GASZ was initially identified by our group as a male and female germ cell and maternal effect gene product [49]. Herein, we show that GASZ is not essential for fertility in the female germline. In contrast, absence of GASZ leads to male sterility due to a block at the zygotene-pachytene transition, reminiscent of the defect that is observed in knockouts of two PIWI family members, MILI and MIWI2 [7],[11],[38]. In frog oocytes, GASZ is expressed in the Balbiani body, a nuage structure [50]. Similarly, we show here that GASZ localizes to nuage in testicular primordial germ cells, gonocytes, spermatogonia, and spermatocytes. Using various markers, GASZ appears to be a component of the nuage termed intermitochondrial cement. We show that many nuage proteins depend upon GASZ for their normal levels. Aside from MIWI, which is expressed in germ cell types absent in the *Gasz* knockout, the reduction of MILI, MAEL, MVH, TDRD1, TDRD6, and TDRD7 in *Gasz^−/−^* testicular lysates likely reflects destabilization of this entire ultrastructural feature. TDRD1 and MVH become mislocalized in gonocytes and they become subsequently lost during postnatal spermatogenesis, suggesting that the loss of nuage proteins may result secondary to degradation following persistent failure to localize to germinal granules. The lesser change in MVH protein in GASZ null testes at P10 may indicate that MVH is less dependent upon GASZ than are the remaining nuage proteins. Perhaps this effect is due to additional proteins functionally redundant with GASZ or that MVH has a greater intrinsic stability in the absence of GASZ than other nuage proteins. Protein-protein interaction motifs in GASZ, including ankyrin domains, a sterile alpha motif, and a leucine zipper, may serve to direct the association and/or stabilization of nuage proteins. GASZ self-interaction may suggest the protein forms multimeric complexes as part of this function. A model depicting direct interactions within the nuage and proposed GASZ placement within this protein complex is provided (Figure S11).

The *Gasz* null mice phenocopy the loss of MILI. Since the TDRD1:MILI interaction is critical [33],[48] and absence of GASZ leads to disruption of the TDRD1 distribution in the cytoplasm (Figure 4), MILI could be physically dislodged from its granule position in *Gasz* null germ cells leading to a subsequent destabilization of MILI. Although formation of intermitochondrial cement is disrupted in both *Tdrd1^−/−^* and *Gasz^−/−^* germ cells, MILI is only unstable in *Gasz* null cells indicating a distinct role for GASZ in stabilization of MILI. As we have shown, that all but 2.2% of germ cells at E16.5 contain MILI, and at P0, MILI is absent. Similar to the *Mili* null mice [6],[11],[38], the absence of MILI in the *Gasz* knockout, is believed to mechanistically disrupt piRNA synthesis, resulting in an increase in retrotransposons and subsequent catastrophe for the male germ line. The current model for maintenance of transposable element repression in the germline involves the production of piRNAs by PIWI family members MILI and MIWI2 in embryonic and postnatal germ cells [6],[7]. Repeat-associated piRNAs are abundant in embryonic germ cells coincident with DNA remethylation of retrotransposons in the male germline at E17.5. GASZ is expressed in embryonic testes and co-localizes with MILI in fetal and newborn gonocytes. Transcriptional regulation of retrotransposons by antisense repeat-associated piRNAs, bound to MIWI2, whose nuclear localization depends upon MILI, has been proposed [13]. Consistent with the proposed mechanism, we observe a similar reduction of promoter methylation of IAP and Line L1 in *Gasz^−/−^* testes as observed in *Mili^−/−^* and *Miwi2^−/−^* testes. Mechanistically, absence of *Gasz* and the consequent loss of MILI and MILI-mediated retrotransposon repression affects fetal gonocytes during the interval that retrotransposon remethylation normally occurs. Although there is a continuity of the cell cycle arrest in male gonocytes between E16.5 and P0, the shift in *Gasz* null testes at E16.5 from slightly detectable MILI to its absence suggests that nuage is changing during this interval despite a lack of cell cycle progression. However, both MVH and TDRD1 displayed similar behavior in *Gasz* null testes at E16.5 and P0. Although we can find a direct interaction between GASZ and MIWI, we could not find evidence for direct interaction between GASZ and MILI. We speculate that a GASZ-MILI interaction occurs indirectly though other mediators. MVH can interact with MILI and MIWI [38], as can TDRD1 [33],[48] both of which co-immunoprecipitate with GASZ. As shown in Figure 5A, GASZ interacts with RANBP9 and MIWI, known MVH interactors. Thus a large complex of proteins may be required for co-localization of GASZ with MILI and its support of MILI function in retrotransposon control. The inability to co-immunoprecipitate GASZ and MILI may suggest that MILI is more weakly “linked” to GASZ in this complex.

Most of the nuage protein mutants that block in meiotic prophase have defects in repeat-associated piRNAs. Nuage protein knockouts cause two types of spermatogenic defects – those that block during meiotic prophase (including MVH, MILI, MIWI2 and MAEL) and those that block during haploid differentiation (including DDX25, MIWI, TDRD1, and TDRD6). In the latter class, *Tdrd1^−/−^* and *Miwi^−/−^* testes do not have altered repeat-associated piRNAs, both of which block at the spermatid stage and do not appear to have defects in stem cell maintenance. Nearly all of the former class possesses some defect in piRNA biosynthesis, but repeat-associated piRNAs are not altered in all mutants of this class. Like GASZ, disruption of MILI and MIWI2 result in an absence of repeat-associated piRNAs and a meiotic block prior to the loss of all germ cells from seminiferous tubules by 6 months of age [6],[11],[38]. The *Mael^−/−^* testis phenotype is unique in showing isolated pachytene piRNA defects but blocking during meiotic prophase with elevated retrotransposons [46]. An important gap in our knowledge is whether *Mvh^1098/1098^* testes display repeat-associated piRNA defects and elevated retrotransposons similar to *vasa* mutants in *Drosophila* [55] and the majority of piRNA pathway mutants blocking during meiotic prophase.

Germ cell apoptosis in *Gasz* null mice may depend upon retrotransposon expression combined with meiotic defects, representing distinct MILI-dependent functions. The GAG, POL, and PRT proteins are all required for efficient retrotransposition of IAP [56], but their individual contribution to cellular toxicity has not been described. Line L1 proteins are toxic to cells by several mechanisms including activation of BAX and caspase 3 [57]. Expression of the reverse transcriptase of ORF2 alone causes cellular pathology in cell lines and could inhibit completion of meiotic recombination in nuage protein knockouts through its ability to bind random breaks in DNA [58],[59]. Alternatively, the cause of apoptosis in *Gasz^−/−^* spermatocytes and nuage knockouts in the meiotic class may be retrotransposon-independent. Two observations suggest MILI regulates meiosis independent of retrotransposons. TDRD1, by preventing the processing of mRNAs into piRNAs, confers specificity in non-repeat piRNA generation by MILI [48]. Line L1 dysregulation in the *Tdrd1^−/−^* mutant must depend upon the decreased mRNAs or increased unannotated RNAs that results from the shift of the substrate RNA used by MILI to generate piRNAs. Since TDRD1 deficient testes do not block during meiotic prophase despite elevated Line L1 expression, spermatocyte death is unlikely to be due to isolated retrotransposon dysfunction. Missense mutants of the zebrafish MILI ortholog ZILI have isolated meiotic defects but no alterations in retrotransposon expression [43], indicating MILI's contribution to meiosis and retrotransposon control are separable. Knockout models suggest that homologous chromosome synapsis depends upon functional MILI [11],[38], MIWI2 [7],[11] and MAEL [46]. *Mvh* null testes block at the zygotene-pachytene transition but their ability to complete synapsis has not been assessed [45]. Therefore, the apoptotic loss of *Gasz^−/−^* spermatocytes may not result from retrotransposon-induced DNA damage alone, but could also result from failed MILI-dependent meiotic functions including homologous chromosome synapsis or meiotic recombination. MILI has been proposed to cause general effects on germ cell mRNA translation not limited to control of retrotransposon mRNAs [37]. It is possible that this aspect of MILI function might contribute to the reduction of nuage protein levels in *Gasz* null testes. Translational control of mRNAs necessary for meiotic prophase by MILI may indirectly affect retrotransposon promoter methylation or post-transcriptional processing of retrotransposon mRNAs in addition to MILI's actions mediated by repeat-associated piRNAs.

The piRNA pathway in mammals is specialized to support male germ cell development. Zebrafish and *Drosophila* share the requirement for PIWI family-dependent piRNA production for fertility of both sexes [42]–[44]; whereas, it is required only for male fertility in the mouse. GASZ is not correlated with this shift because GASZ orthologs are present in many vertebrates including zebrafish [50]. In the mammalian male germline, GASZ supports piRNA biosynthesis required for initiation of retrotransposon repression during the embryonic period and maintenance during meiotic prophase when alterations to chromatin and transcriptional increase would otherwise be favorable toward their expression. GASZ is the first mammalian germline- and intermitochondrial cement-specific protein lacking domains for RNA modification which impacts piRNA processing by localizing or stabilizing multiple proteins in the nuage including PIWI family members.

## Materials and Methods

### Generation of *Gasz* Mutant Mice and Fertility Studies

We electroporated the linearized *Gasz* targeting vector (Figure S1A) into the HPRT-negative AB2.2 ES cell line; selected clones in hypoxanthine, aminopterine, thymidine, and 1-(2 -deoxy-2 fluoro-D-arabinofuranosyl)-5-iodouracil; and screened the ES cell DNA by Southern blot as described [60] to identify the mutant Gasz allele, *Gasz* ^tm1Zuk^ (herein called *Gasz^−^*). Correctly targeted clones were identified by using 5′ and 3′ probes as shown. Targeted ES cell clones were injected into blastocysts to produce chimeric male mice [61], which were bred to produce C57BL6/J×129 hybrid F1 *Gasz* ^−/−^ offspring. Ten homozygous mutant and heterozygous sires were bred with WT females over a 6-month mating period. Similar mating trials were performed with homozygous mutant and heterozygous dams. Except for testis defects, *Gasz^−/−^* mice were grossly indistinguishable from their littermates and lived to become adults.

### Generation of the Anti-GASZ Antibody and Western Blot Analysis

Full-length His-tagged GASZ was injected into guinea pigs to produce polyclonal antibodies (Cocalico Biologicals, Reamstown, PA). Membranes with 20 or 50 µg of total testis lysate per lane were probed with guinea pig anti-GASZ (1∶1000), rabbit anti-IAP GAG (1∶1000), rabbit anti-TDRD1 (1∶1000), rabbit anti-TDRD6 (1∶1000), rabbit anti-TDRD7 (1∶500), rabbit anti-MVH (1∶500), rabbit anti-MAELSTROM (1∶250 Abcam ab28661), or rabbit anti-MILI (1∶250 Abcam ab36764) polyclonal antibodies. After developing, the membrane was stripped and re-probed with anti-β-actin clone AC-15 (Sigma) at 1∶5,000. Secondary anti-guinea pig and anti-mouse horseradish peroxidase-conjugated antibodies (Jackson ImmunoResearch, West Grove, PA) were used at 1∶10,000.

### Immunohistochemistry, Immunofluorescence, and Co-Immunoprecipitation Western Analysis

Testes were fixed in Bouin's fixative (for histology), 4% paraformaldehyde (immunofluorescence) or 4% paraformaldehyde/6.6% acetic acid (for immunohistochemistry), and embedded in paraffin. Antigen retrieval was performed on 5 µm sections by boiling for 20 minutes in citrate buffer pH 6.0. Samples for immunofluorescence were incubated with rabbit anti-γH2AX (1∶5,000; 05–636 Upstate, now Millipore, Billerica, MA), guinea pig anti-GASZ (1∶300), rabbit anti-MVH (1∶500), rabbit anti-TDRD1 (1∶100), rabbit anti-MILI (1∶500), rabbit anti-IAP GAG (1∶500), rabbit anti-ORF1 (1∶500), and mouse anti-cytochrome c (1∶300, 556433 BD Biosciences San Jose, CA). Alexa594-conjugated anti-guinea pig, Alexa488-conjugated anti-rabbit, and Alexa488-conjugated anti-mouse antibodies (Jackson ImmunoResearch) were used at 1∶500. Sections were mounted with Vectashield mounting medium with DAPI or propidium iodide (Vector Laboratories). Representative images for immunofluorescence were selected and captured on a Zeiss Axiovert s100 2TV. When required, deconvolution was performed with softWoRx v3.3.6 (Applied Precision, Issaquah, WA).

One mg of testicular lysates from WT mice were prepared with 0.5% NP-40 lysis buffer as described previously [62]. Pre-cleared lysates were incubated overnight at 4°C with anti-GASZ, anti-MIWI, or no primary antibody, followed by incubation with protein G beads in 5% BSA to precipitate immune complexes. Co-immunoprecipitating proteins were detected by western blotting with anti-MVH (1∶500; ab13840 Abcam Inc, Cambridge, MA), anti-MIWI (1∶1000), anti-TDRD1 (1∶1000), anti-MVH (1∶500; Abcam Cambridge, MA), anti-GASZ (1∶500), or β actin as above. Secondary antibodies used were horseradish peroxidase-labeled donkey anti-rabbit, goat anti-mouse, or goat anti-guinea pig (Jackson ImmunoResearch, West Grove, PA). Ten µg of WT and age-matched *Gasz^−/−^* lysates were used as loading controls.

### Meiotic Marker and Apoptosis Evaluation

To evaluate meiotic progression, slides were incubated with guinea pig anti-GASZ (1∶500), rabbit anti-SYCP3 (1∶500), or rabbit anti-H1.T (1∶1000). Staining was visualized using biotinylated goat anti-rabbit or goat anti-mouse secondary antibodies at 1∶200 and the Vectastain ABC Kit according to the manufacturer (Vector Laboratories, Burlingame, CA). TUNEL analysis was performed with three sections from five mice of each genotype for 10-, 21-day-old and 6-week-old mice by using the Chemicon ApopTag Fluorescein In Situ Apoptosis Detection Kit (S7110). Representative images were captured on a Ziess Axioskop (Carl Zeiss MicroImaging, Thornwood, NY).

### Yeast Two-Hybrid Screening

Yeast two-hybrid screening was performed by using CLONTECH Matchmaker Two-Hybrid Library Construction & Screening Kit. A yeast cDNA library was constructed from 17-day-old mouse testis cDNA. Full-length mouse *Gasz* cDNA, was subcloned into pGBKT7 vector, and was used as the bait construct for screening a yeast cDNA library constructed from 17-day-old mouse testis cDNA. After yeast mating, clones that grew on (SD Leu- Trp- Ade- His- X-α-Gal) selection plates were isolated, and candidate pGADT7-Rec-cDNAs were sequenced. Interactions were confirmed by mating mouse *Gasz* bait with prey constructs.

To analyze how GASZ interacts with itself and other interacting proteins we generated prey constructs, by using pGADT7, expressing full-length GASZ, MILI, MIWI, and MVH constructs, as well as a RANBP9 construct lacking its proline-rich amino terminus (aa 1–50). Using the CLONTECH Matchmaker BioSensor Kit All constructs were confirmed by DNA sequencing. We made the mating culture by using full-length mouse *Gasz* bait mated with the prey constructs. Protein-protein interactions were quantified from the resultant fluorescent signal of an equal number of mated cells on a 96 well oxygen biosensor plate post culture for 24 hours using the CLONTECH Matchmaker BioSensor Kit. We added an equal number of mated cells to a 96 well oxygen biosensor plate, cultured the cells at 30°C for 24 hours, and quantified the resultant fluorescent signal.

### RT-PCR and QPCR

Using the Superscript III Reverse Transcriptase Kit (Invitrogen, Rockville, MD) cDNA was synthesized from total mouse testis RNA from mice on embryonic day 18.5 and postnatal days 0, 7, and 14 primed with random hexamers following treatment with Turbo DNA-free (Ambion, Austin, TX). *Gasz* was PCR amplified using ACCGGTCCTCTCAGAAATTAAAA (forward, 111–133 of NM_023729.2) and ATTGGCGTCATAAGTCTCCTACA (reverse, 455–477 of NM_023729.2) primers. QPCR was performed in duplicate with primers specific to IAP and LINE1 [7] on six 14-day-old animals of each genotype using SYBR Green PCR Master Mix on a 7500 Real Time PCR System machine (Applied Biosystems). Nuage protein mRNAs were similarly quantified using primers designed in Primer Express (Applied Biosystems, Foster City, CA) with the exception of those for Mvh [63] and are described in Table S1. Relative quantification was performed using *Actb* or *Gapdh* as appropriate. Cycle conditions were as follows: one cycle at 50°C for 2 min, followed by 1 cycle at 95°C for 10 min, followed by 40 cycles at 95°C for 15 s and 60°C for 1 min. The relative amount of transcripts was calculated by the ΔΔCT method. SEM was calculated for the duplicate measurements, and the relative amount of target gene transcripts was plotted. Significance was determined using Student t-test.

### Electron Microscopic Analysis of Nuage

Testes were removed from newborn mice and drop fixed in a modified Karnovsky's mixture of 2.5% glutaraldehyde, 2% paraformaldehyde plus 2 mM CaCl_2_ in 0.1 M cacodylate buffer pH 7.4 overnight at 4°C. After primary fixation, the tissue was rinsed 3 times for 5 minutes in 0.1 M cacodylate buffer pH 7.4. The tissue was then post-fixed in 1% OsO_4_ in pH 7.4. The tissue was dehydrated in a gradient series from 30% to 100% ethanol. After ethanol dehydration, the tissue was given 3 changes of fresh propylene oxide for 20 minutes each. The tissue was infiltrated up to 1∶1 propylene oxide plus Spurr's Low Viscosity embedding resin. The tissue was placed in individual 00 BEEM capsules and polymerized at 70°C overnight. Thick sections were cut with a HistoDiatome knife on an RCM MT-6000XL ultra-microtome, stained with toluidine blue and examined on a light microscope for orientation. Thin sections were cut at 80 nm using a Diatome Ultra knife on the same ultra-microtome and picked up on 200 mesh copper grids. Thin sections were stained for 15 minutes in a saturated aqueous solution of uranyl acetate, counterstained for 6 minutes with Reynold's lead citrate and examined on a Hitachi H7500 transmission electron microscope. Digital images were captured using Gatan Digital Micrograph software and a Gatan US1000 camera.

### Methylation Analysis of LINE L1 and IAP

Total testicular genomic DNA was recovered from 3 pooled 14-day-old mice by phenol:chloroform extraction and ethanol precipitation. Aliquots of DNA were denatured for 15 minutes at 50°C in 0.3 M NaOH and bisulfite treatment was performed as described in [64]. Briefly, samples were incubated at 50°C overnight with sodium metabisulfite and hydroxyquinone and purified using a modified procedure for a Viral RNA Miniprep kit (Qiagen). Samples were desulfonated in NaOH for 15 minutes, neutralized with HCl, and purified as before. PCRs were performed using Platinum Taq Supermix (Invitrogen) or ExTaq (Takara) using primers described below from [11] to amplify twelve CG dinucleotides in IAP LTR1_Mm ERVK and from [7] to amplify eight CG dinucleotides in L1MD-A2. Each PCR was performed in 4 replicates, which were pooled and purified using a DNA clean-up and concentrator kit (Zymo Research). Amplicons were ligated into pGEM-T Easy vector (Promega) and sequenced. Capital letters in the reference sequences below denote sequences that are complementary to primer binding sites. These do not contain CG dinucleotides, and all cytosine residues are expected to convert during bisulfite treatment. Conversion of essentially all cytosines not part of CG dinucleotides confirmed the efficacy of bisulfite treament.

IAP LTR1_Mm ERVK (prebisulfite conversion): chr10:83255039–83255316 (all IAP sequence)CTGTGTTCTAAGTGGTAAACAAATAATCTGcgcatgtgccaagggtatcttatgactacttgtgctctgccttccccgtgacgtcaactcggccgatgggctgcagccaatcaaggagtgacacgtccgaggcgaaggagaatgctccttaagagggacggggttttcgtttttctctctctcttgcttcgctctctcttgcttcttgctctcttttcctgaagatgtaagaataaagctttgccgcagaagATTCTGGTCTGTGGTGTTCTTCCTG

Forward primer IAP-bisF2 from: TTGTGTTTTAAGTGGTAAATAAATAATTTG

Reverse primer IAP-bisR2 from: CAAAAAAAACACACAAACCAAAAT

L1Md-A2 (prebisulfite conversion): chrX:154580101-1544585723 (L1 beginning at 154580138)AAGTTACAAATAATTTTCTGGGGCCcggatctggggcacaagtcccttccgctcgactcgtgactcgagccccgggctaccttgccagcagagtcttgcccaacacctgcaagggtccacacaggactccccgcgggaccctaagacctctGGTGAGTGGATCACAGTGCCTGCCC

Forward primer methyl L1-F: AAGTTATAAATAATTTTTTGGGGTT

Reverse primer methyl L1-R: AAACAAACACTATAATCCACTCACC

### Small RNA Isolation, Sequencing, and Bioinformatics Analysis

Fifteen µg of total RNA from WT and *Gasz^−/−^* mouse testes were gel-fractionated to isolate 18–40 nt small RNAs, followed by 3′ and 5′ adapter ligation, and product amplification by RT-PCR as per the small RNA kit (FC-102-1009, Illumina) protocol. Finally, the small RNA library was sequenced using a Solexa/Illumina GA-1 Genome analyzer. Small RNA sequences were analyzed through a high-throughput computational pipeline. For each sample, all sequence reads were aligned to a reference set of miRNAs (miRNA pipeline) and all currently identified piRNAs. The reads are also mapped to the reference mouse genome (NCBI Build 37, UCSC mm9) using the Pash software package [65],[66], and uploaded to Genboree platform (www.genboree.com) to identify snoRNAs, scRNAs and repeat-associated small RNAs. We performed a local Smith-Waterman alignment of each unique sequence read against each of the mature microRNAs in miRBase version 11.0, allowing for a 3 base overhang on the 5′ end and a 6 base overhang on the 3′ end. The alignments were scored such that a matching or overhanging base counts as 2 points and mismatches as −1. Each unique sequence read which achieves a per-base alignment score of 2 (i.e., a perfect match) was associated with each mature microRNA for which it achieved that score. The read counts of all redundantly aligning reads to multiple hairpins in the genome were equally apportioned to each mature microRNA to which they align. For repeat-associated small RNAs, each mapping is associated with LINEs, SINEs, DNA and RNA repeat subtypes. Reads were also mapped to consensus mouse and mammalian repeats from Repbase [67], and rasiRNAs [11] using Blat [68] with a sensitive setting only requiring one 8mer seed filtered such that≥90% of the reads mapped. All sequences corresponding to repeat-associated piRNAs and putative novel piRNAs have been deposited at piRNABank (http://pirnabank.ibab.ac.in/) [69].

## Supporting Information

Figure S1 Histological analysis of 5-day-old testes. Histological analysis of testes of *Gasz^+/−^* and *Gasz^−/−^* mice. Seminiferous tubules for both genotypes contain spermatogonia and juvenile Sertoli cells. G, spermatogonia; Mit, mitotically dividing spermatogonia; Ser, Sertoli cells. [Scale bar: 20 µm]
(7.69 MB TIF)

Figure S2 The XY body fails to appear in *Gasz^−/−^* testes due to elevated DNA damage. (A–C) γH2AX localizes in WT testes to perinuclear chromatin of early germ cells [arrow in (A)] and the XY body in pachytene spermatocytes [arrowhead in (B)] while there is increased non-XY body staining in *Gasz^−/−^* [arrow in (C)]. [Scaling: 5,000×magnification]
(2.87 MB TIF)

Figure S3 Immunohistochemical and TUNEL analysis of juvenile *Gasz* ^+/−^ and *Gasz* ^−/−^ testes. (A–D) Immunohistochemical analysis of testes of *Gasz^+/−^* and *Gasz^−/−^* mice using antibodies to SYCP3 and H1.T. SYCP3, a marker for all primary spermatocytes [70], labeled fewer spermatocytes in *Gasz^−/−^* than *Gasz^+/−^* testes from 12-day-old mice. H1.T is a testis-specific histone H1 expressed at low levels in early spermatocytes, peaking in late pachytene spermatocytes, and continued expression in round spermatids [71]. While numerous H1.T-positive spermatocytes were detected in *Gasz^+/−^* testes, few H1.T-positive cells were observed in *Gasz^−/−^* testes composed of predominantly early spermatocytes and rare atypical “pachytene” spermatocytes. (E–J) TUNEL analysis was performed on the *Gasz^+/−^* and *Gasz^−/−^* testes. In *Gasz^+/−^* testes, TUNEL-positive germ cells were rare, predominantly affecting spermatogonia with the exception of 21-day-old testes when there is a normal developmental peak in germ cell apoptosis [72]. In *Gasz^−/−^* testes at postnatal days 10 and 21 and in the adult, there is enhanced germ cell apoptosis. By their size, location, and abundance, the most dying cells appear to be spermatocytes. Consistent with pachytene spermatocyte loss being restricted to the stages I-VI of the cycle of the seminiferous epithelium (Figure S5), after 14 days of age we observed more inter-tubule variation including tubules lacking TUNEL positive cells. [Scale bars: 100 µm (A–D) and 50 µm (E–J)]
(9.80 MB TIF)

Figure S4 GASZ localizes to perinuclear cytoplasmic granules. Immunolocalization of GASZ in adult testes using anti-GASZ antibody. Immunostaining is detectable in spermatogonia [G in (B,F)], preleptotene spermatocytes [PL in (C)], pachytene spermatocytes [P in (A–D)], and round spermatids [R in (A–C)]. Staining of leptotene [L in (D)] and zygotene spermatocytes [Z in (E)] as well as elongating [E in (E–F)] and condensing [C in (A–C)] spermatids was negligible. The most intense staining was detected in middle to late pachytene spermatocytes where GASZ displays a granular distribution pattern in the perinuclear region of the cytoplasm. Only dying germ cells are immunoreactive in *Gasz^−/−^* seminiferous tubules (G). STAGE I, V, VIII, IX, X, XII in (A–F) designates the corresponding stage seminiferous tubule.
(9.51 MB TIF)

Figure S5 Diagramatic summary of *Gasz* ^−/−^. Loss of pachytene spermatocytes in *Gasz^−/−^* testes correlates with the stages of the seminiferous epithelium where GASZ immunostaining is most intense (green bars). Pachytene spermatocytes in stages I–VI (yellow) can be seen undergoing apoptosis. The most mature germ cells in stage VII–XII seminiferous tubules are early spermatocytes. All germ cells absent from *Gasz^−/−^* testes are shown in red. The diagram is modified from [73].
(0.70 MB TIF)

Figure S6 IWI and GASZ co-localize in late pachytene spermatocytes. Staining is shown for GASZ [(A) in green], MIWI [(B) in red], and merge (C). GASZ and MIWI co-localize in some granules in pachytene spermatocytes (arrows). GASZ does not co-localize with MIWI in the chromatoid body (arrowheads). [Scaling: 5,000×magnification]
(2.48 MB TIF)

Figure S7 MVH levels are reduced in *Gasz* null spermatocytes. Immunofluorescent analysis of *Gasz^+/−^* (A) and *Gasz^−/−^* (B) testes. MVH prominently stains spermatocytes in *Gasz^+/−^* testes [asterisks in (A)] versus low level staining of spermatogonia in *Gasz^−/−^* testes [arrowheads in (B)]. [Scaling: 5,000×magnification]
(1.67 MB TIF)

Figure S8 Nuage marker mRNAs are modestly reduced in embryonic and juvenile *Gasz^−/−^* testes. Quantitative RT-PCR analysis of MIWI2, MILI, MVH, TDRD1, MAEL, and MIWI in testes from e18.5, 7-, and 14-day-old mice (mean±SEM). (A) *Gasz^−/−^* embryonic testes show no alteration of nuage mRNAs. (B) In the postnatal testis most nuage markers are not significantly reduced until post-natal day 14 (P14) with the exception of MIWI2 mRNA which was reduced at post-natal day 7 (P7).
(0.43 MB TIF)

Figure S9 Intermitochondrial cement is absent from *Gasz^−/−^* gonocytes. Electron micrographs depicting a nuage localized to clustered mitochondria in *Gasz^+/−^* [arrowheads in (A,C,E)] and the lack of a corresponding structure in *Gasz^−/−^* newborn testes (B,D,F).
(4.99 MB TIF)

Figure S10 Length and nucleotide composition analysis of repeat-associated and unknown small RNAs. Comparison of small RNA length in control and *Gasz^−/−^* testes from 10-day-old mice that mapped with ≥90% identity using Blat to consensus elements including all repeats (A), LTRs (B), LINE L1s (C), and SINEs (D). Developmental abundance of small RNA classes in *Gasz^−/−^* testes and controls at postnatal days 7 (P7), 10 (P10), and 14 (P14) including SINE-associated small RNAs (E), repeat-associated small RNAs (19–23 nt), and other repeat-associated small RNAs. (H–I) Compositional analysis of the 1^st^ and 10^th^ nucleotides of repeat-associated piRNAs (25–29 nt) (H) and repeat-associated small RNAs (19–23 nt) (I). (E,F) Characterization of length of the unknown category of small RNAs (J), developmental abundance of the unknown small RNAs (19–23 nt) in *Gasz^−/−^* testes and controls, and comparison of the nucleotide composition for 25–29 nt versus the 19–23 nt classes (F).
(1.22 MB TIF)

Figure S11 Model for GASZ interaction with nuage proteins. A summary of reported interactions between nuage proteins suggests that they may form a protein network containing GASZ. Physical interactions between nuage proteins are depicted by connecting lines.
(0.09 MB TIF)

Table S1 Primers for quantitative RT-PCR assessment of nuage components.
(0.02 MB XLS)

Table S2 Non-repeat-associated piRNAs are reduced in *Gasz* ^−/−^ testes. The average number of reads for piRNAs from two *Gasz^−/−^* (KO) and control testes from postnatal day 7, 10, and 14 (P7, P10, and P14) mice are shown along with their percentage of the total reads from each testis. All piRNAs that were absent or reduced greater than three-fold in *Gasz^−/−^* from controls are highlighted in gray. piRNAs with greater abundance on D7 and D14 were analyzed separately. piRNA ID is the accession assigned by piRNABank (http://pirnabank.ibab.in) followed by the Genbank accession. The fold-change represents the degree of reduction in *Gasz^−/−^* from controls and was calculated for age-matched samples.
(17.58 MB XLS)

Table S3 miRNAs missannotated as piRNAs. Sequences annotated as piRNAs that increased in abundance in *Gasz^−/−^* testes were compared to the map positions (UCSC mm9) of mouse pre-miRNAs. The majority of overlapping sequences mapped to a large microRNA cluster on the X chromosome. The “piRNA” reads observed and the similar pre-miRNA with mature miRNA sequences in bold are provided. Most of the sequences are identical with mature miRNAs or differ by 1–2 nt at the 3′ end. We also observed several piRNAs mapping to the strand of the stem-loop opposite to known miRNAs to which we assigned putative designations. Novel testis piRNA t27 is described in [74].
(0.03 MB XLS)

Table S4 A subset of repeat-associated small RNAs are reduced in *Gasz* ^−/−^ testes. The average number of individual small RNAs from two *Gasz* ^−/−^ (KO) and control testes from postnatal day 7 (P7), 10 (P10), and 14 (P14) mice that mapped with greater than 90% identity to individual repeat consensuses are shown along with their percentage of the total reads from each testis. Each repeat is classified according to a class of repeats (LINE, SINE, LTR) and families within that class. The fold reduction of piRNAs in the *Gasz* ^−/−^ as compared to controls is shown with those elements reduced 10-fold highlighted in gray. Among those repeats displaying the most dramatic sustained reduction in *Gasz* null mice included LTRs (LTR45, MER72B, MER87, RLTR10, LTR75, LTR90B, MT2_Mm, MamGyp-Int, MT-Int, MTB-Int, ORR1A0, ORR1A-Int, ORR1A1, and several IAPs) and LINE L1s (L1MD_A, L1Md_F3, L1Md_Gf, Lx2A1). SINE-associated small RNAs, processed by Dicer, are mostly unaffected in *Gasz* ^−/−^ testes. SR (Simple Repeat); LC (Low Complexity); UNK (Unknown classification).
(0.56 MB XLS)
