# Correction: GASZ Is Essential for Male Meiosis and Suppression of Retrotransposon Expression in the Male Germline

**DOI:** 10.1371/annotation/20bf08d1-07e9-451e-b079-166832ebe158

**Published:** 2009-12-30

**Authors:** Lang Ma, Gregory M. Buchold, Michael P. Greenbaum, Angshumoy Roy, Kathleen H. Burns, Huifeng Zhu, Derek Y. Han, R. Alan Harris, Cristian Coarfa, Preethi H. Gunaratne, Wei Yan, Martin M. Matzuk

The following information was omitted from the Funding section: These studies were supported in part by the Eunice Kennedy Shriver NICHD/NIH through cooperative agreement U01-HD060496 (to MMM).

