# Supplementary figures and images for: GASZ Is Essential for Male Meiosis and Suppression of Retrotransposon Expression in the Male Germline
Source: PLoS Genet. 2009 Sep 4;5(9):e1000635. doi: 10.1371/journal.pgen.1000635 (PMC2727916; doi:10.1371/journal.pgen.1000635)

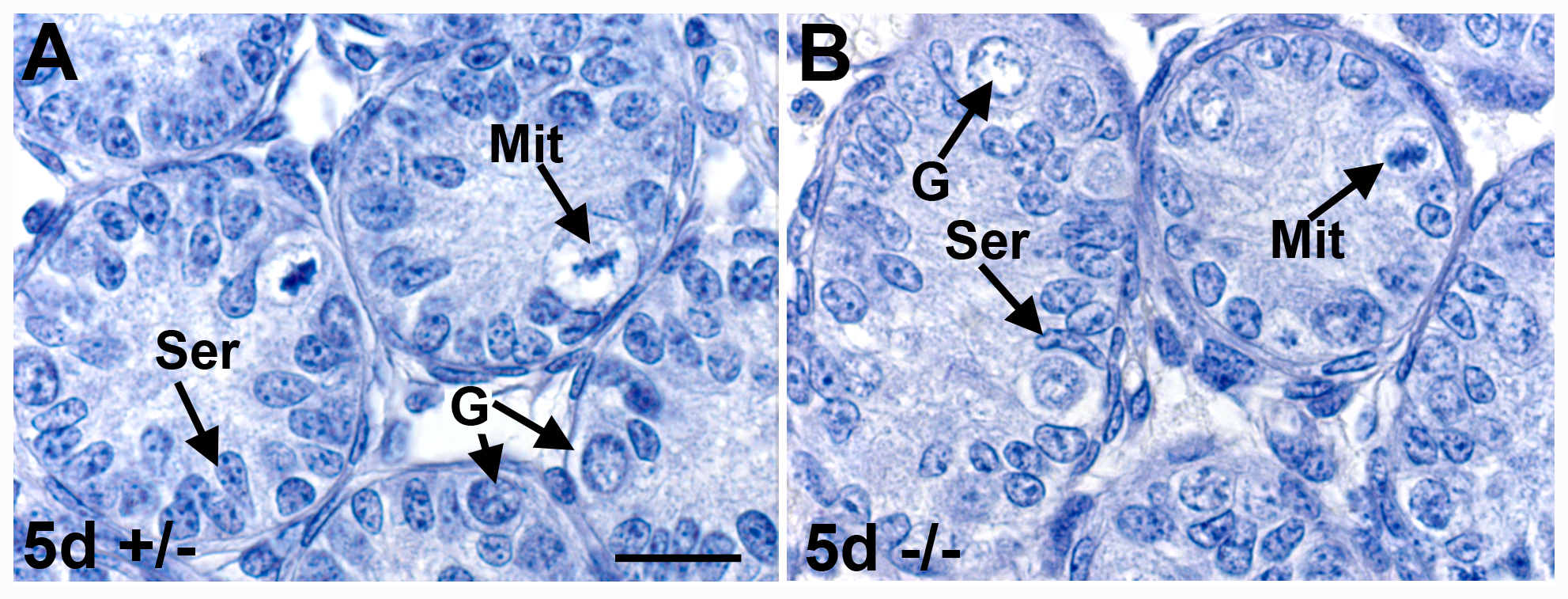

Supplement: Figure S1 — Histological analysis of 5-day-old testes. Histological analysis of testes of Gasz+/− and Gasz−/− mice. Seminiferous tubules for both genotypes contain spermatogonia and juvenile Sertoli cells. G, spermatogonia; Mit, mitotically dividing spermatogonia; Ser, Sertoli cells. [Scale bar: 20 µm] (7.69 MB TIF) [file pgen.1000635.s001.tif]

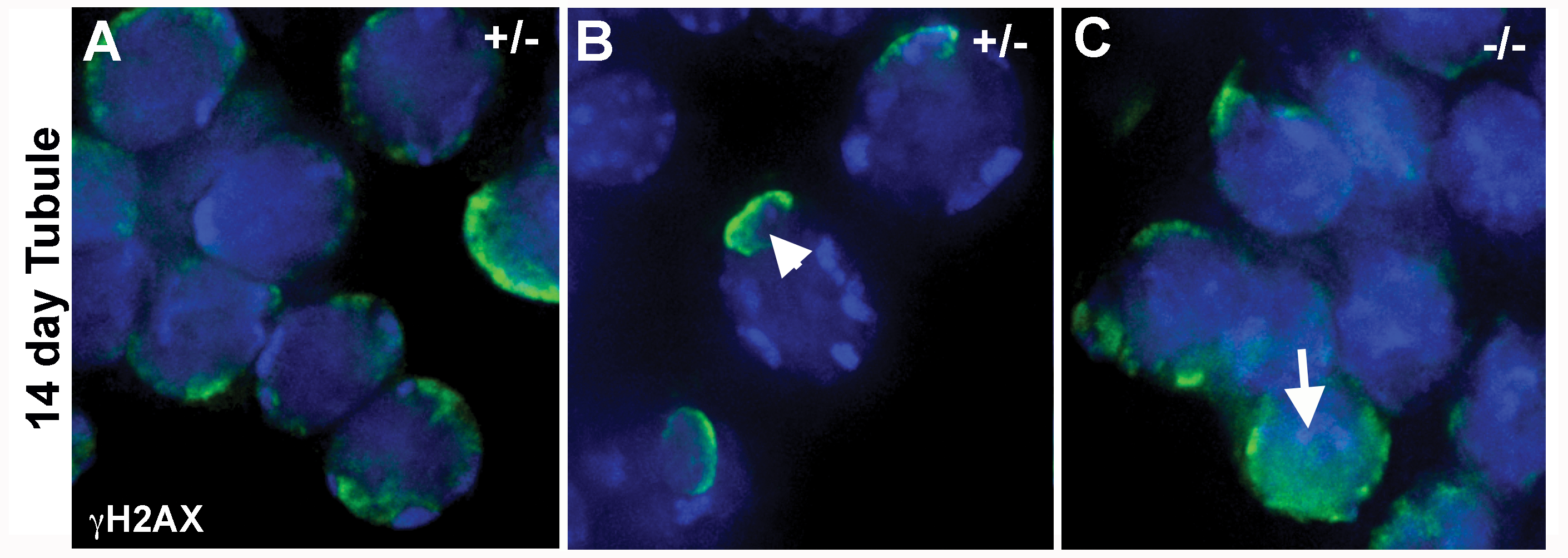

Supplement: Figure S2 — The XY body fails to appear in Gasz−/− testes due to elevated DNA damage. (A–C) γH2AX localizes in WT testes to perinuclear chromatin of early germ cells [arrow in (A)] and the XY body in pachytene spermatocytes [arrowhead in (B)] while there is increased non-XY body staining in Gasz−/− [arrow in (C)]. [Scaling: 5,000×magnification] (2.87 MB TIF) [file pgen.1000635.s002.tif]

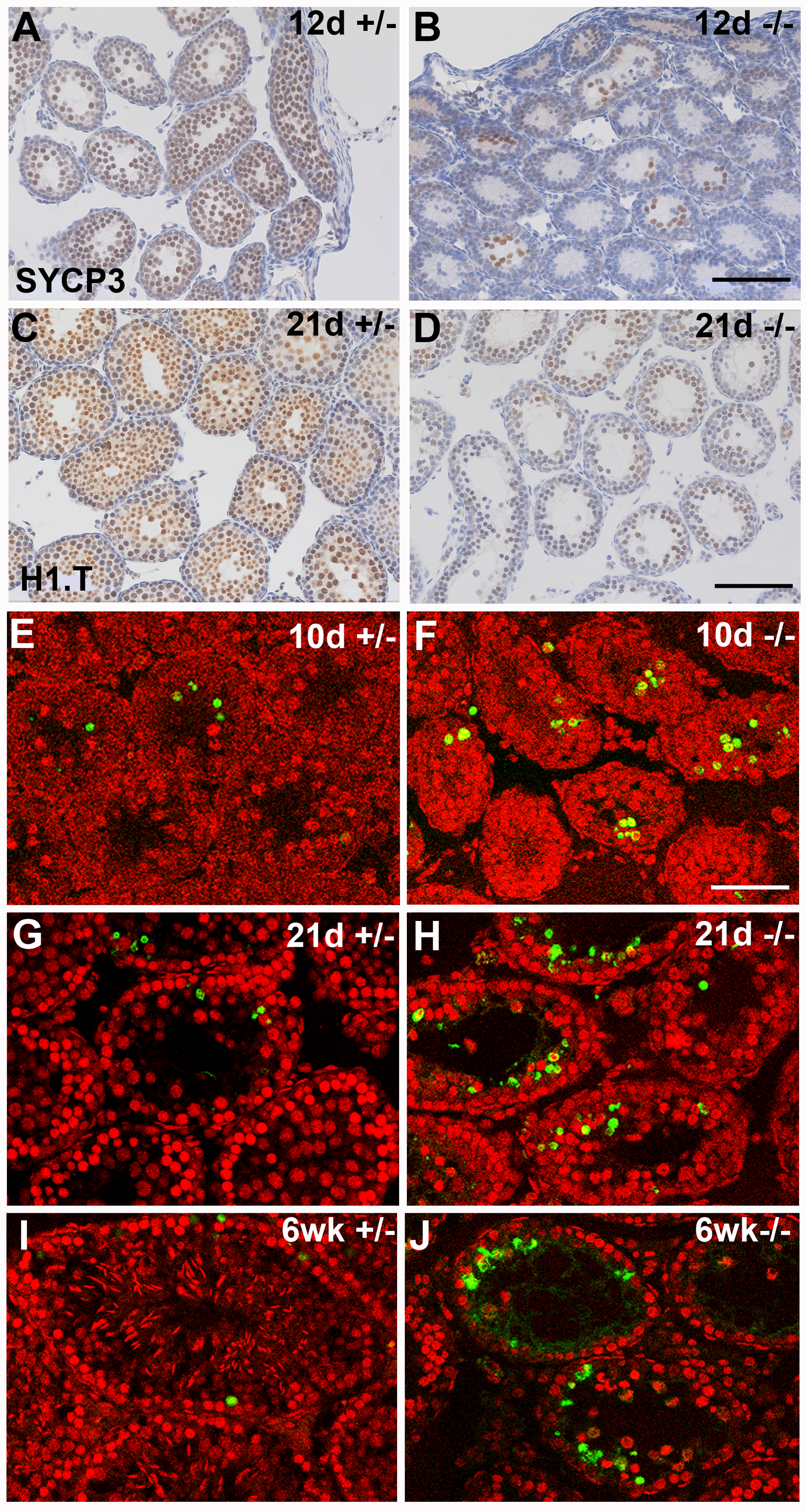

Supplement: Figure S3 — Immunohistochemical and TUNEL analysis of juvenile Gasz +/− and Gasz −/− testes. (A–D) Immunohistochemical analysis of testes of Gasz+/− and Gasz−/− mice using antibodies to SYCP3 and H1.T. SYCP3, a marker for all primary spermatocytes [70], labeled fewer spermatocytes in Gasz−/− than Gasz+/− testes from 12-day-old mice. H1.T is a testis-specific histone H1 expressed at low levels in early spermatocytes, peaking in late pachytene spermatocytes, and continued expression in round spermatids [71]. While numerous H1.T-positive spermatocytes were detected in Gasz+/− testes, few H1.T-positive cells were observed in Gasz−/− testes composed of predominantly early spermatocytes and rare atypical “pachytene” spermatocytes. (E–J) TUNEL analysis was performed on the Gasz+/− and Gasz−/− testes. In Gasz+/− testes, TUNEL-positive germ cells were rare, predominantly affecting spermatogonia with the exception of 21-day-old testes when there is a normal developmental peak in germ cell apoptosis [72]. In Gasz−/− testes at postnatal days 10 and 21 and in the adult, there is enhanced germ cell apoptosis. By their size, location, and abundance, the most dying cells appear to be spermatocytes. Consistent with pachytene spermatocyte loss being restricted to the stages I-VI of the cycle of the seminiferous epithelium (Figure S5), after 14 days of age we observed more inter-tubule variation including tubules lacking TUNEL positive cells. [Scale bars: 100 µm (A–D) and 50 µm (E–J)] (9.80 MB TIF) [file pgen.1000635.s003.tif]

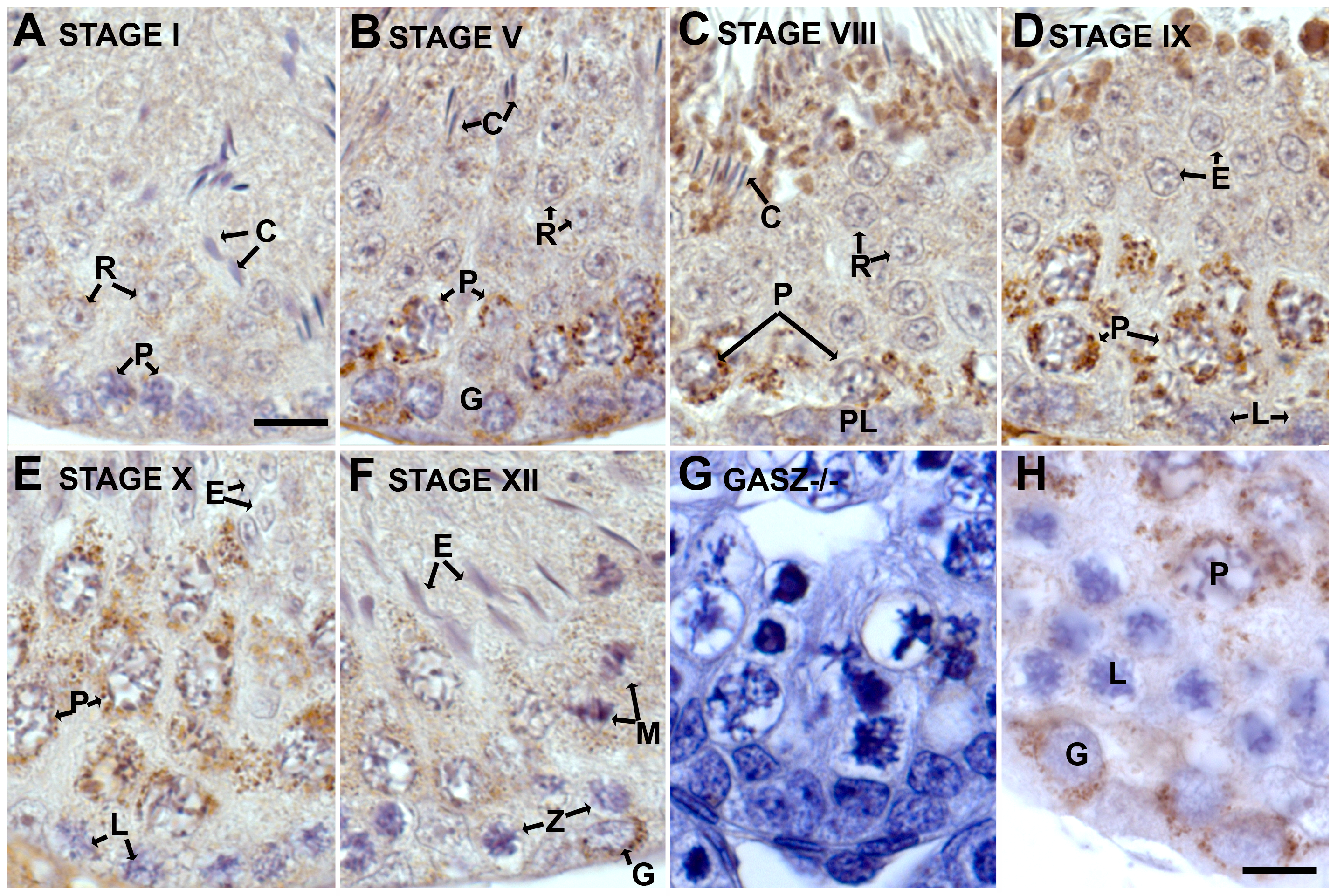

Supplement: Figure S4 — GASZ localizes to perinuclear cytoplasmic granules. Immunolocalization of GASZ in adult testes using anti-GASZ antibody. Immunostaining is detectable in spermatogonia [G in (B,F)], preleptotene spermatocytes [PL in (C)], pachytene spermatocytes [P in (A–D)], and round spermatids [R in (A–C)]. Staining of leptotene [L in (D)] and zygotene spermatocytes [Z in (E)] as well as elongating [E in (E–F)] and condensing [C in (A–C)] spermatids was negligible. The most intense staining was detected in middle to late pachytene spermatocytes where GASZ displays a granular distribution pattern in the perinuclear region of the cytoplasm. Only dying germ cells are immunoreactive in Gasz−/− seminiferous tubules (G). STAGE I, V, VIII, IX, X, XII in (A–F) designates the corresponding stage seminiferous tubule. (9.51 MB TIF) [file pgen.1000635.s004.tif]

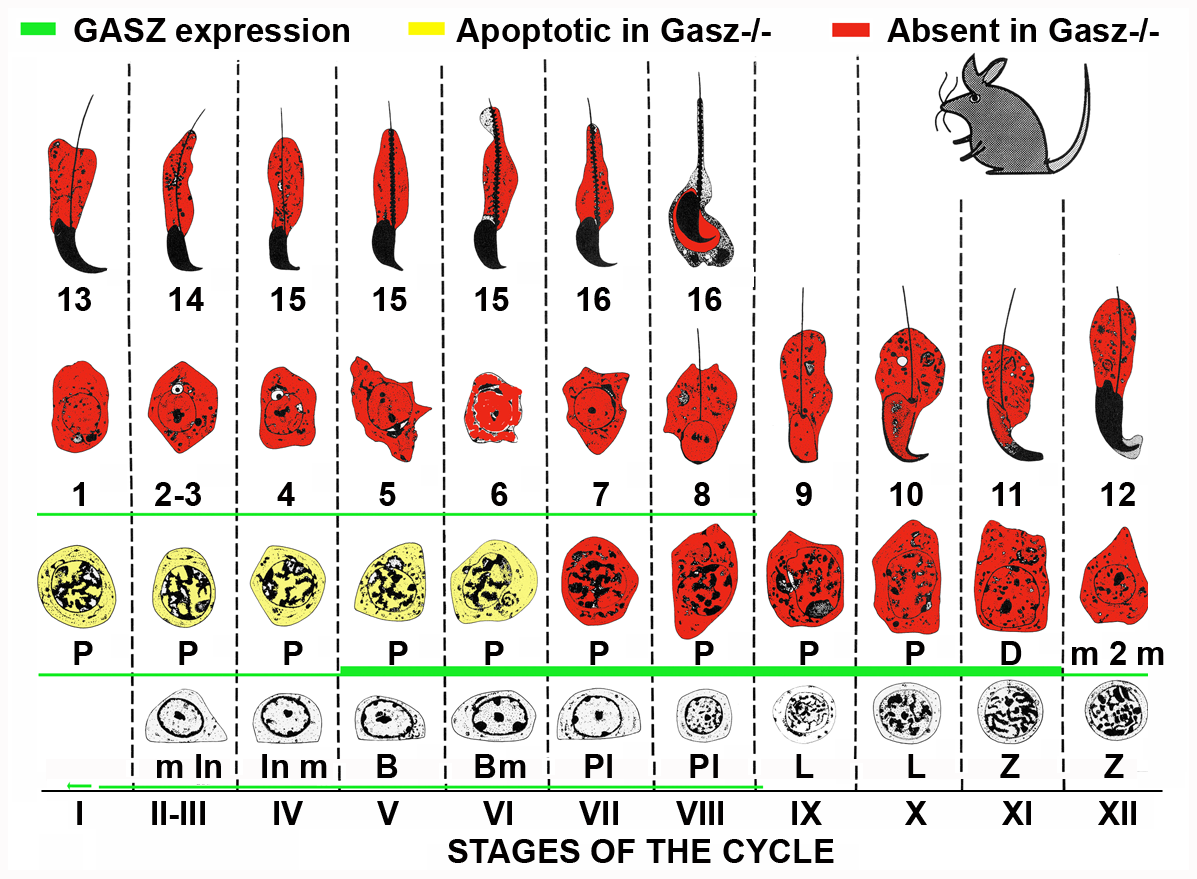

Supplement: Figure S5 — Diagramatic summary of Gasz −/−. Loss of pachytene spermatocytes in Gasz−/− testes correlates with the stages of the seminiferous epithelium where GASZ immunostaining is most intense (green bars). Pachytene spermatocytes in stages I–VI (yellow) can be seen undergoing apoptosis. The most mature germ cells in stage VII–XII seminiferous tubules are early spermatocytes. All germ cells absent from Gasz−/− testes are shown in red. The diagram is modified from [73]. (0.70 MB TIF) [file pgen.1000635.s005.tif]

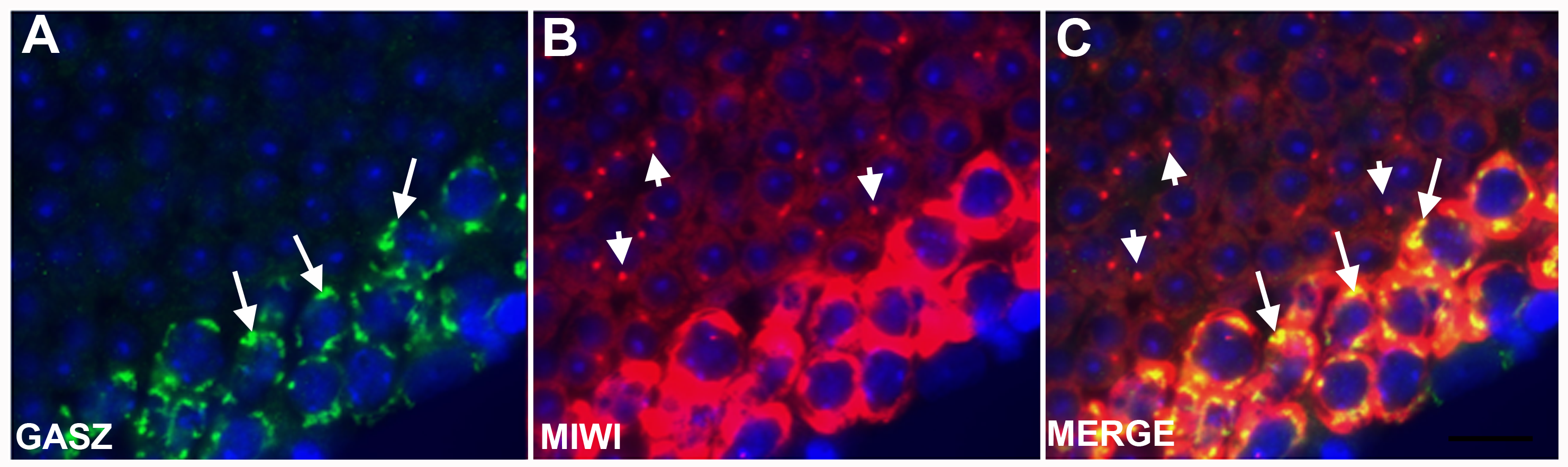

Supplement: Figure S6 — IWI and GASZ co-localize in late pachytene spermatocytes. Staining is shown for GASZ [(A) in green], MIWI [(B) in red], and merge (C). GASZ and MIWI co-localize in some granules in pachytene spermatocytes (arrows). GASZ does not co-localize with MIWI in the chromatoid body (arrowheads). [Scaling: 5,000×magnification] (2.48 MB TIF) [file pgen.1000635.s006.tif]

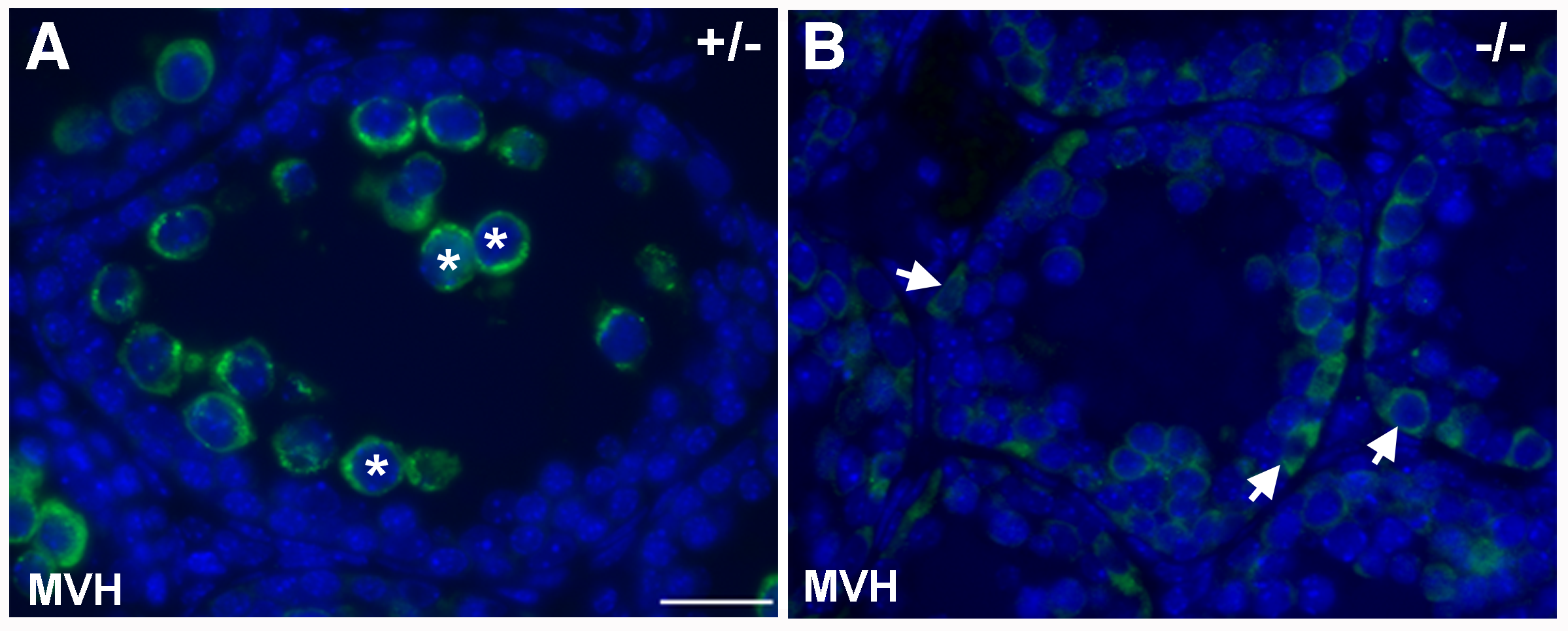

Supplement: Figure S7 — MVH levels are reduced in Gasz null spermatocytes. Immunofluorescent analysis of Gasz+/− (A) and Gasz−/− (B) testes. MVH prominently stains spermatocytes in Gasz+/− testes [asterisks in (A)] versus low level staining of spermatogonia in Gasz−/− testes [arrowheads in (B)]. [Scaling: 5,000×magnification] (1.67 MB TIF) [file pgen.1000635.s007.tif]

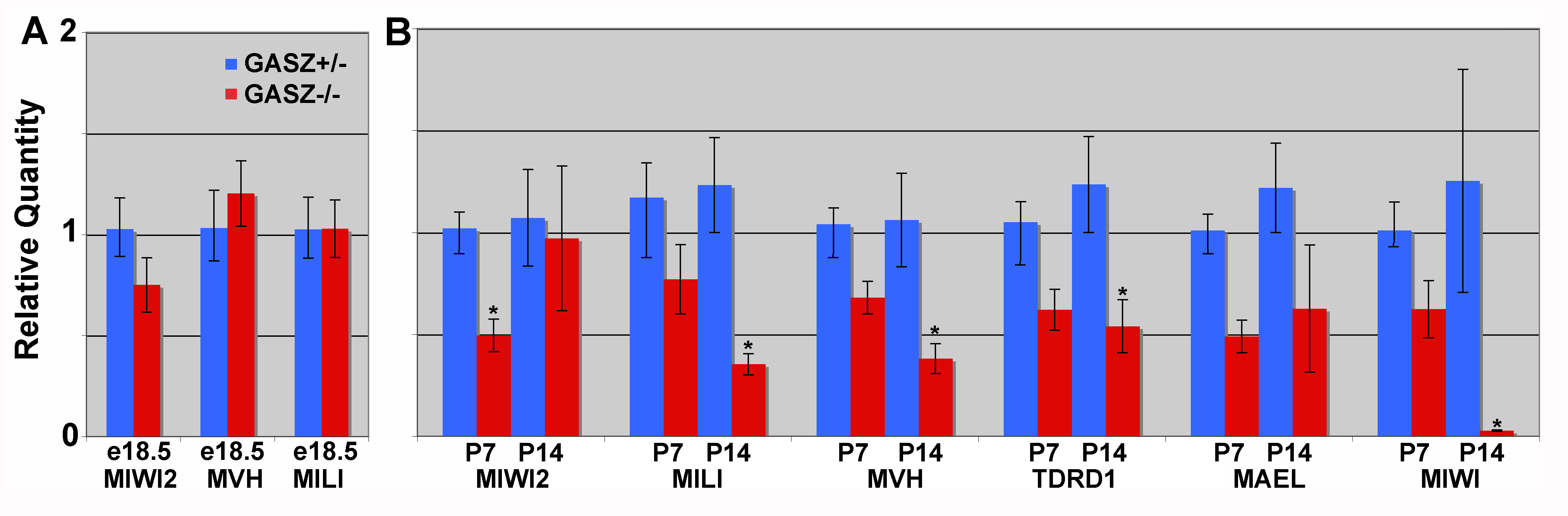

Supplement: Figure S8 — Nuage marker mRNAs are modestly reduced in embryonic and juvenile Gasz−/− testes. Quantitative RT-PCR analysis of MIWI2, MILI, MVH, TDRD1, MAEL, and MIWI in testes from e18.5, 7-, and 14-day-old mice (mean±SEM). (A) Gasz−/− embryonic testes show no alteration of nuage mRNAs. (B) In the postnatal testis most nuage markers are not significantly reduced until post-natal day 14 (P14) with the exception of MIWI2 mRNA which was reduced at post-natal day 7 (P7). (0.43 MB TIF) [file pgen.1000635.s008.tif]

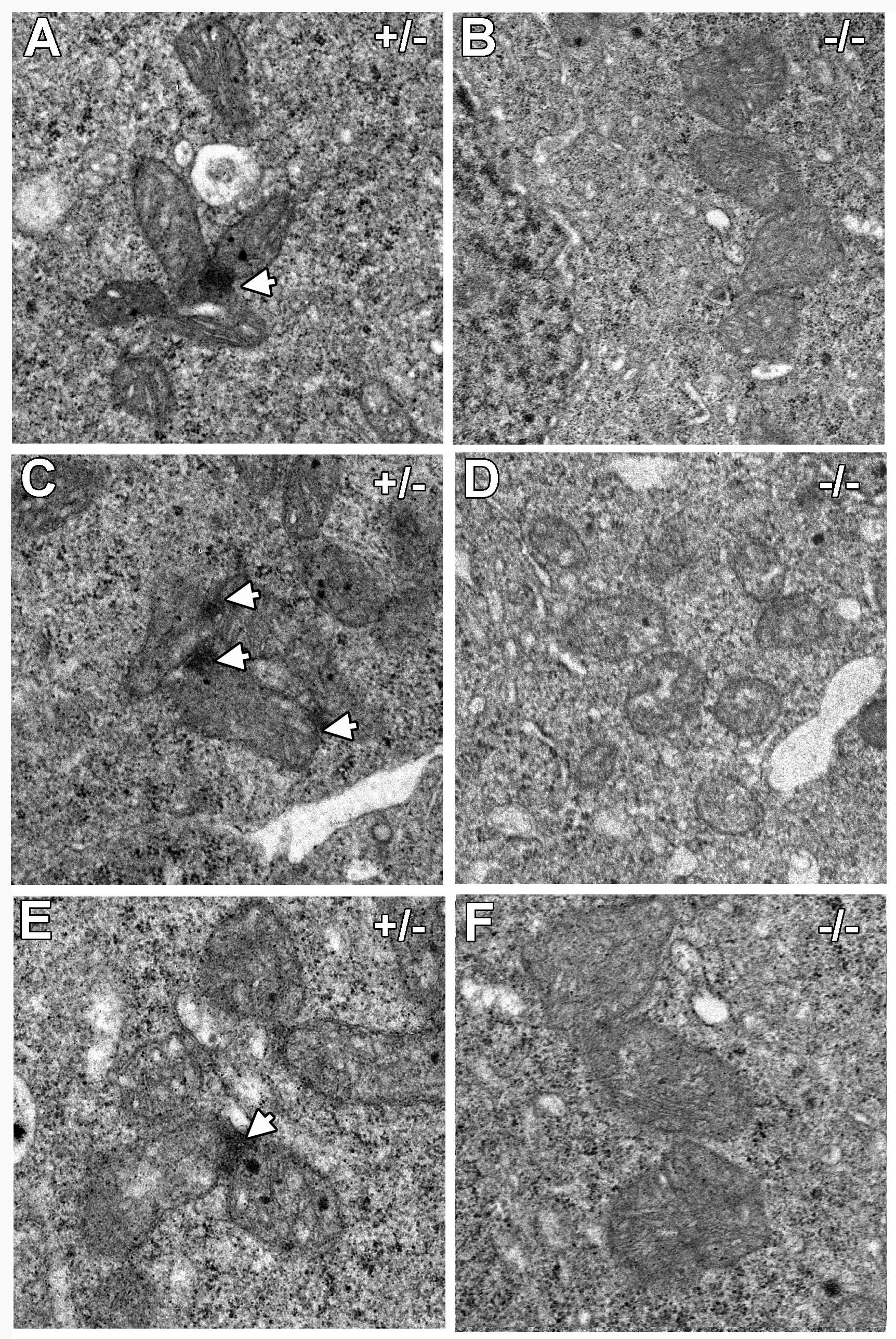

Supplement: Figure S9 — Intermitochondrial cement is absent from Gasz−/− gonocytes. Electron micrographs depicting a nuage localized to clustered mitochondria in Gasz+/− [arrowheads in (A,C,E)] and the lack of a corresponding structure in Gasz−/− newborn testes (B,D,F). (4.99 MB TIF) [file pgen.1000635.s009.tif]

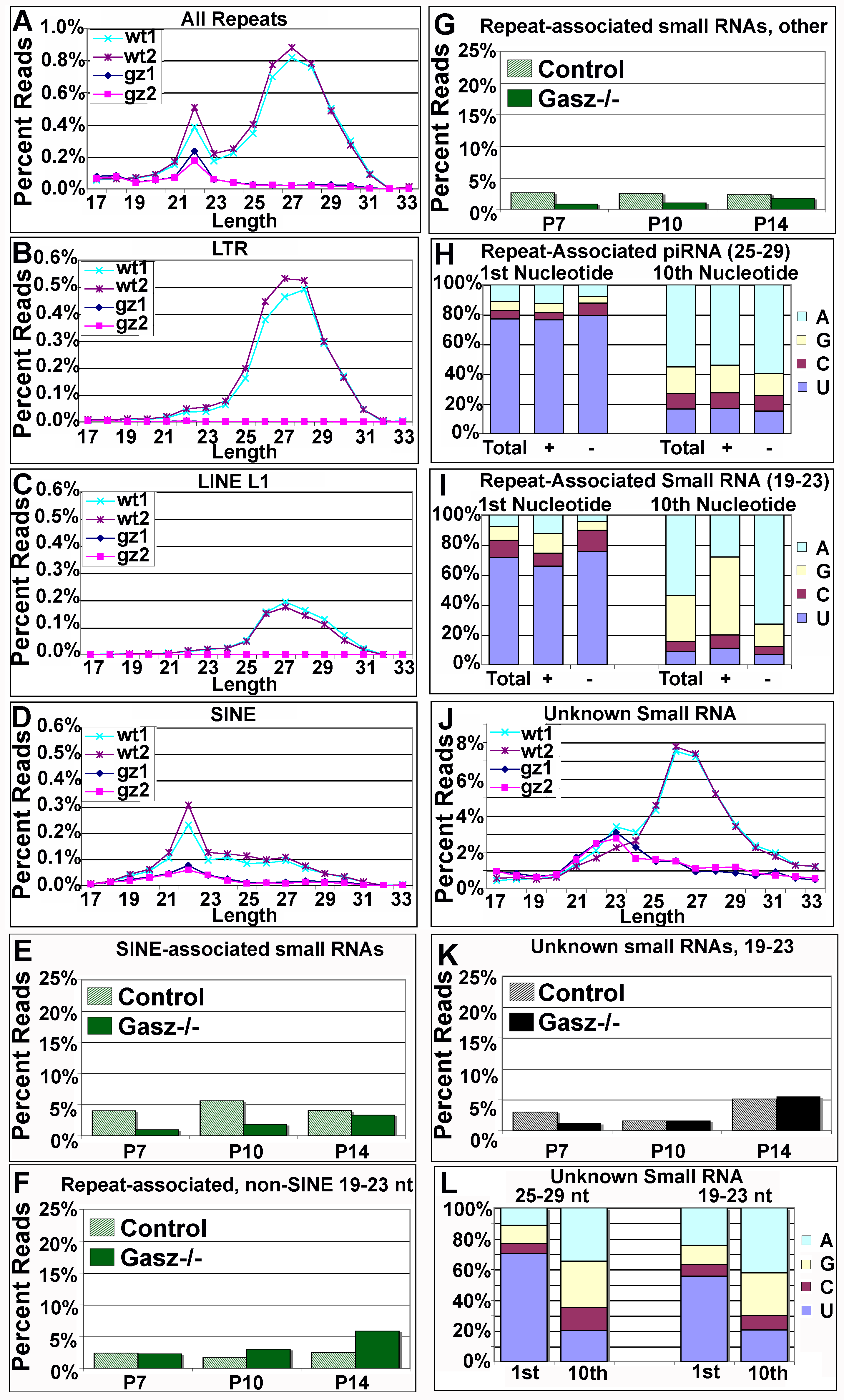

Supplement: Figure S10 — Length and nucleotide composition analysis of repeat-associated and unknown small RNAs. Comparison of small RNA length in control and Gasz−/− testes from 10-day-old mice that mapped with ≥90% identity using Blat to consensus elements including all repeats (A), LTRs (B), LINE L1s (C), and SINEs (D). Developmental abundance of small RNA classes in Gasz−/− testes and controls at postnatal days 7 (P7), 10 (P10), and 14 (P14) including SINE-associated small RNAs (E), repeat-associated small RNAs (19–23 nt), and other repeat-associated small RNAs. (H–I) Compositional analysis of the 1st and 10th nucleotides of repeat-associated piRNAs (25–29 nt) (H) and repeat-associated small RNAs (19–23 nt) (I). (E,F) Characterization of length of the unknown category of small RNAs (J), developmental abundance of the unknown small RNAs (19–23 nt) in Gasz−/− testes and controls, and comparison of the nucleotide composition for 25–29 nt versus the 19–23 nt classes (F). (1.22 MB TIF) [file pgen.1000635.s010.tif]

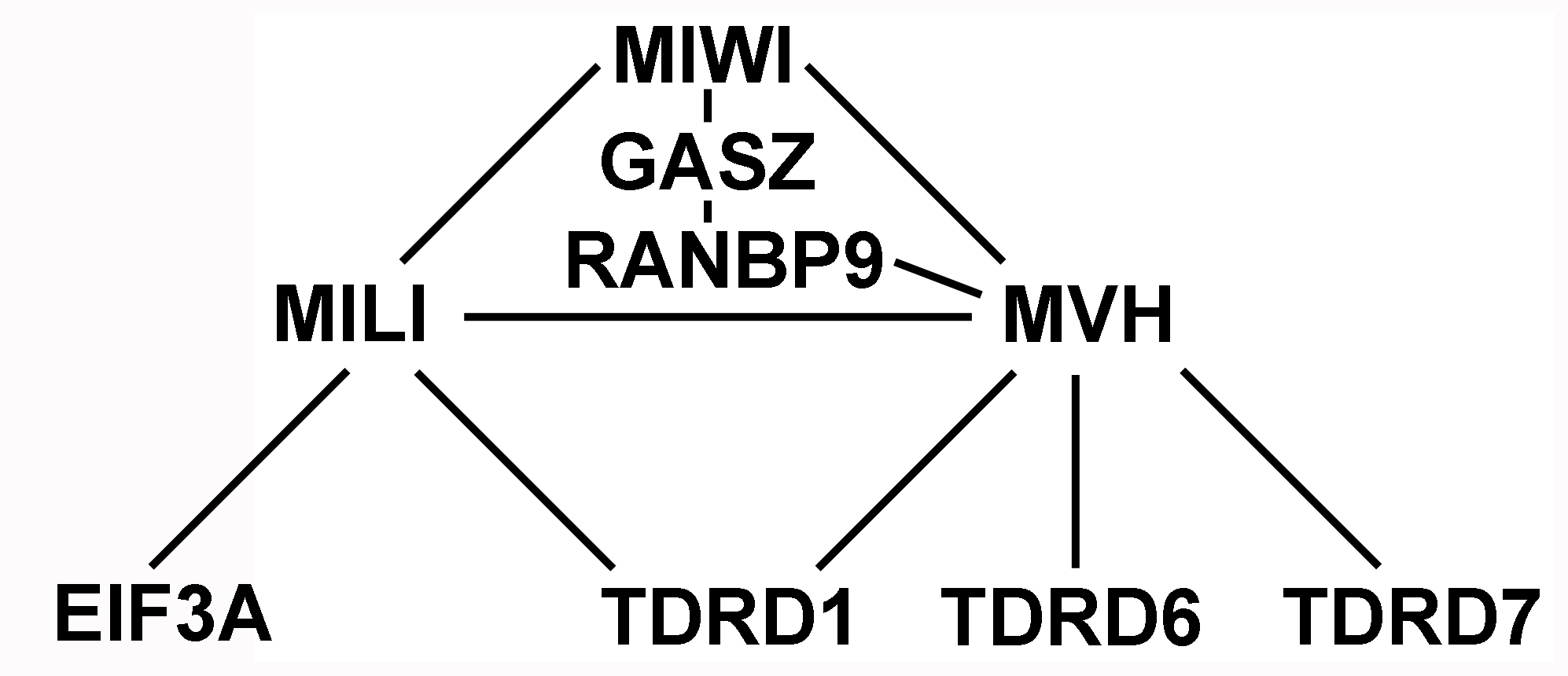

Supplement: Figure S11 — Model for GASZ interaction with nuage proteins. A summary of reported interactions between nuage proteins suggests that they may form a protein network containing GASZ. Physical interactions between nuage proteins are depicted by connecting lines. (0.09 MB TIF) [file pgen.1000635.s011.tif]
